# Long-Acting HIV-1 Fusion Inhibitory Peptides and their Mechanisms of Action

**DOI:** 10.3390/v11090811

**Published:** 2019-09-02

**Authors:** Chen Wang, Shuihong Cheng, Yuanyuan Zhang, Yibo Ding, Huihui Chong, Hui Xing, Shibo Jiang, Xuebing Li, Liying Ma

**Affiliations:** 1 State Key Laboratory of Infectious Disease Prevention and Control, National Center for AIDS/STD Control and Prevention, Collaborative Innovation Center for Diagnosis and Treatment of Infectious Diseases, Chinese Center for Disease Control and Prevention, Beijing 102206, China; 2 CAS Key Laboratory of Pathogenic Microbiology and Immunology, Institute of Microbiology, Chinese Academy of Sciences, Beijing 100101, China; 3 MOH Key Laboratory of Systems Biology of Pathogens, Institute of Pathogen Biology and Center for AIDS Research, Chinese Academy of Medical Sciences and Peking Union Medical College, Beijing 100730, China; 4 Key Laboratory of Medical Molecular Virology (MOE/NHC/CAMS), School of Basic Medical Sciences, Fudan University, Shanghai 200032, China

**Keywords:** HIV-1, long-acting fusion inhibitory peptide, antiviral activity

## Abstract

The clinical application of HIV fusion inhibitor, enfuvirtide (T20), was limited mainly because of its short half-life. Here we designed and synthesized two PEGylated C34 peptides, PEG2kC34 and PEG5kC34, with the PEG chain length of 2 and 5 kDa, respectively, and evaluated their anti-HIV-1 activity and mechanisms of action. We found that these two PEGylated peptides could bind to the HIV-1 peptide N36 to form high affinity complexes with high α-helicity. The peptides PEG2kC34 and PEG5kC34 effectively inhibited HIV-1 Env-mediated cell–cell fusion with an effective concentration for 50% inhibition (EC_50_) of about 36 nM. They also inhibited infection of the laboratory-adapted HIV-1 strain NL4-3 with EC_50_ of about 4–5 nM, and against 47 HIV-1 clinical isolates circulating in China with mean EC_50_ of PEG2kC34 and PEG5kC34 of about 26 nM and 32 nM, respectively. The plasma half-life (t_1/2_) of PEG2kC34 and PEG5kC34 was 2.6 h and 5.1 h, respectively, and the t_1/2_ of PEGylated C34 was about 2.4-fold and 4.6-fold longer than C34 (~1.1 h), respectively. These findings suggest that PEGylated C34 with broad-spectrum anti-HIV-1 activity and prolonged half-life can be further developed as a peptide fusion inhibitor-based long-acting anti-HIV drug for clinical use to treat HIV-infected patients who have failed to respond to current anti-retrovirus drugs.

## 1. Introduction

Approximately 36.7 million people are living with HIV, with 20.9 million having access to antiretroviral therapy (ART) [1]. ART has been successful in controlling HIV-1 replication, especially in suppressing HIV loads, restoring immune function, as well as improving longevity and quality of life [2]. Yet, steps should be taken to develop more drugs based on the very rapid emergence of drug resistance and the poor pharmacokinetics of drugs [3,4,5].

HIV fusion inhibitors, which block the fusion of the viruses into cells to finally reduce cellular damage, have become a research focus. In a generally accepted viral fusion model, the viral envelope surface glycoprotein gp120 binds sequentially to cell receptor CD4 and a coreceptor (CCR5 or CXCR4), triggering conformational changes in the core structure of gp41 [6], which, as determined by X-ray crystallography, reveals a stable 6-helix bundle (6-HB) consisting of 3 central N-terminal heptad repeat (NHR) coiled coils and 3 C-terminal heptad repeat (CHR) helices packing into the hydrophobic NHR grooves as antiparallel [7]. In the past two decades, discovery of HIV-1 fusion inhibitory peptides from the NHR and CHR regions of gp41 opened an avenue for developing antiviral agents [8,9,10]. Although many HIV-1 anti-fusion peptides reported showed ideal antiviral activity, their short half-life and unavoidable drug resistance make HIV-1 fusion inhibitors still insufficient. Now, structural modification of existing drugs has become an important way to obtain new drugs. Polyethylene glycol (PEG) conjugation, as a commonly used method for improving the pharmacokinetics of therapeutic peptides and proteins, has been successfully applied to design drugs [11,12,13]. After an initial experiment, a 2 kDa PEG and a 5 kDa PEG were conjugated to the N-terminus of C34 to improve the pharmaceutical profiles in this study. 

In this study, we have successfully synthesized two long-acting HIV-1 fusion inhibitory peptides: PEGylated C34. We confirmed their binding to NHR to form stable 6-HB, then analyzed their inhibitory activity on Env-mediated cell–cell fusion and virus infection and tested their pharmacological kinetics in rats.

## 2. Materials and Methods 

### 2.1. Ethics

We complied with relevant institutional and national standards (laboratory animal use license: SYXK 2014-0032) for animal care and experimentation. This study was reviewed by ethics community of the Institute of Microbiology of the Chinese Academy of Sciences (No.SQIMCAS2019012) in March 2019. The TZM-bl and MT2 cell lines were obtained from National Institutes of Health (NIH) AIDS Research and Reference Reagent Program. The HIV-1 viruses used in the infectivity assays were isolated and replicated in the “European Research Infrastructures for Poverty Related Diseases” project. The informed consent was signed before the sample collection. This study was reviewed by the Institutional Research Ethics Community of Chinese Center for Disease Control and Prevention (No.201334).

### 2.2. Peptide Design Synthesis and Identification

The peptide of C34 was synthesized using a solid-phase Fmoc method at Beijing Scilight Biotechnology LLC (Beijing, China), purified by HPLC (purity >95%), and characterized by MALDI−TOFmass spectra. We investigated the site-specific addition of PEG to the terminus of C34 using a thiol-maleimide coupling reaction, following our established glycosylation strategy [12,14]. Briefly, PEG2kC34 and PEG5kC34 were prepared using conjugation derivatives that have a cysteine residue at the N-termini of C34 (cC34), as well as the PEG derivations modified with methoxy-PEG-maleimides (mPEG-MAL) of 2 kDa and 5 kDa. An aqueous solution of disodium hydrogen phosphate (5 mM) was added in a dropwise manner to a stirred mixture of cysteine-incorporated C34 (C34, 10 mg, 2.3 µmol) and mPEG-MAL (2 or 5 kDa, 2 equivalent) in a sodium phosphate buffer (5 mL, 5 mM, pH 7.5) to adjust the pH of the mixture to 7.2. The resulting mixture was then stirred for 10 min at room temperature before being directly purified by preparative HPLC to afford PEG2kC34 (12 mg, 82%) and PEG5kC34 (15 mg, 70%), as a white powder. HPLC analyses were performed on an Agilent 1200 system (California, USA) equipped with a UV detector. The HPLC chromatographic conditions were as follows: Agilent C8 column (4 mm, 9.4 × 250 mm), 40→70% CH3CN/H2O linear gradient at 2.0 mL/min, UV detection at 210 nm. MALDI-TOF mass spectra were recorded on a Bruker BIFLEX III spectrometer (Bremer, Germany).

### 2.3. Circular Dichroism (CD) Spectroscopy

The N36 peptide (10 nM) was incubated with an equal molar concentration of C peptide at 37 °C for 30 min in phosphate-buffered saline (PBS; pH 7.2). CD spectroscopy were acquired on a Jasco spectropolarimeter (Model J-815), using a 1 nm band width with a 1 nm step resolution from 195 to 270 nm at room temperature. The α-helical content was calculated from the CD signal by dividing the mean residue ellipticity at 222 nm. Thermal denaturation was performed by monitoring the ellipticity change at 222 nm from 20 °C to 98 °C at a rate of 1.2 °C/min [15,16].

### 2.4. Determination of gp41 6-HB Formation by Native Polyacrylamide Gel Electrophoresis (N-PAGE)

Tris-glycine gel (18%) was used for N-PAGE. Peptide N36 was incubated with PBS at the indicated concentrations at 37 °C for 30 min before the addition of C34, PEG2kC34 and PEG5kC34 (final concentration of the peptides, 100 μM). The gradient concentration of the peptide N36 was 0, 50 and 100 μM, respectively. After incubation for another 30 min at 37 °C, the samples were mixed with Tris-glycine native sample buffer at a volume ratio of 4:1, and the mixtures were then loaded onto the well of a gel (25 μL for each well). Gel electrophoresis was carried out at a constant voltage of 120 V at room temperature for 3–4 h. The gel was stained with Coomassie blue and visualized with an imaging system [15].

### 2.5. Cytotoxicity Assay

The cytotoxicity of the peptides to TZM-bl cells was measured. Briefly, 100 μL of serially diluted peptide with the initial concentration of 100 μM was added to 100 μL overnight cultured cells (1 × 10^4^/well) on a 96-well plate. After incubation at 37 °C for 2 days, 20 μL of the original concentration CCK-8 reagent (DOJINDO, Japan) was added to the cells. After incubation for 2 h, the absorbance at 450 nm and 630 nm was measured using a BioTek Epoch 2 ELISA reader (Vermont, USA) at room temperature. The 50% cytotoxicity concentrations (CC_50_) were calculated as previously described [2].

### 2.6. Inhibition of HIV-1 Env-Mediated Cell–Cell Fusion

The inhibitory activity of the peptides on HIV-1 Env-mediated cell–cell fusion was determined. Briefly, HEK-293T cells were seeded at 5 × 10^5^ cells/well in six well plates for overnight incubation. Then, 1.0 μg pcDNA3.1-Tat plasmid and 0.5 μg pNL4-3-Env plasmid were transfected into HEK-293T cells with the Invitrogen lipofectamine 3000 kit (California, USA) according to the instructions. The next day, TZM-bl cells expressing CD4 and the coreceptor for HIV-1 as target cells were seeded at 1 × 10^4^ in DMEM medium containing 10% fetal bovine serum (FBS) in wells of a 96-well plate. On the third day, 100 µL of peptide at graded concentrations was added to the 96-well plate. Then, HEK293T cells, which were transferred with plasmids encoding the HIV-1 Env, as the effector cells, were added to TZM-b1 cells at a ratio of 1:1. The percent inhibition of a peptide against HIV-1 Env-mediated cell–cell fusion was calculated using the luciferase assay [17]. The EC_50_ was calculated as previously described [2].

### 2.7. Inhibition of HIV-1 Replication

Inhibition of HIV-1 replication was measured by infection assay as previously described in [2]. Briefly, HIV-1 culture supernatants were harvested, and 50% tissue culture infectious doses (TCID_50_) were determined in TZM-bl cells. Different initial concentrations were set depending on the EC_50_ of the peptide. Then peptides were diluted in DMEM medium in 2-fold dilutions and added to the TZM-bl cells (10^4^/well), followed by addition of HIV-1 (200 TCID_50_/well). After culture for 48 h at 37 °C, the viral infectivity was measured using the luciferase assay and the inhibitory activity of the peptides was calculated.

### 2.8. Pharmacokinetics of Peptides in Rats

To examine the pharmacological kinetics in vivo, Sprague Dawley^®^ (SD) rats were used for the collection of plasma as reported [14,18,19]. The animal experiment was conducted under ethical guidelines and approved by the Institutional Laboratory Animal Care and Use Committee at the Chinese Academy of Sciences. Briefly, SD rats (*n* = 4, 2 male and 2 female, 7 weeks) were given a single subcutaneous injection of C34, PEG2kC34 or PEG5kC34 at a dosage of 1.7 μmol/kg in physiological saline, to which a small amount of borax buffer at pH 9.5 was added as a solubilization agent. Blood samples (300 μL) were harvested from the tail vein before injection and at different time intervals after injection (0.5, 1.0, 2.0, 4.0, 6.0, 8.0, 10.0 and 24.0 h). All blood samples were added into microtubes, which contained a small amount of sodium heparin and aprotinin. Plasma samples were collected by centrifugation of the whole-blood samples (4500 g/min for 10 min at 4 °C) and were tested for ex vivo anti-HIV-1 activity as previously described [18]. The highest dilution-fold of the plasma sample causing 50% inhibition of HIV-1 infection was calculated, based on which the concentration of an active peptide in plasma was estimated [20] and its half-life and other pharmacokinetic parameters were calculated using MODFIT software [21]. 

## 3. Results

### 3.1. Generation of High-Purity PEGylated HIV-1 Fusion Inhibitory Peptide

**Figure 1:**
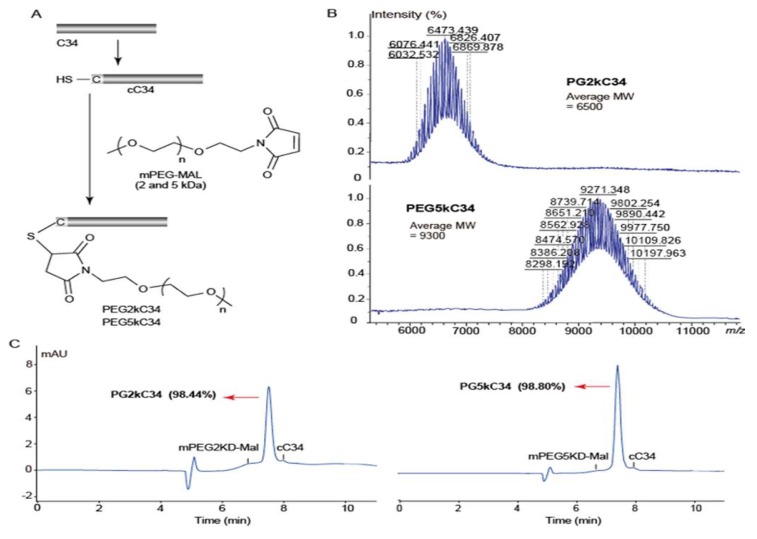
Peptide design, synthesis and identification. Sequence of C34: WMEWDREINNYTSLIHSLIEESQNQQEKNEQELL. The peptide sequences were protected by N-terminal acetylation and C-terminal amidation. (**A**) The flowchart of synthesis PEG2kC34 and PEG5kC34. Reaction conditions: Phosphate buffer (pH 7.2), room temperature, 10 min. (**B**) MALDI-TOF mass spectrum analysis of the molecular weight of PEG2kC34 and PEG5kC34. (**C**) HPLC chromatogram analysis of the purity of PEG2kC34 and PEG5kC34. HPLC analyses were performed on an Agilent 1200 system (Agilent, USA) equipped with a UV detector. HPLC chromatographic conditions: Agilent C8 column (4 mm, 9.4 × 250 mm), 40→70% CH_3_CN/H_2_O linear gradient at 2.0 mL/min, UV detection at 210 nm.

Based on previous work [22], C-terminal modification results in decreased antiviral activity, so the site-specific modification of C34 was compiled at its N-terminus with PEG2kD and PEG5kD(Figure 1A), by using our developed strategies [12]. The reaction proceeded in mild conditions (pH 7.2, room temperature) and finished within a short period of time (<10 min) to afford nearly quantitative PEGylated C34. The products were purified by semi-preparative HPLC and characterized by MALDI-TOF mass spectrum (Figure 1B). The high purity (>98%) of the product was obtained as white powder for further bioactivity study (Figure 1C).

### 3.2. Binding of PEGylated C34 to NHR Forms Stable 6-HB 

**Figure 2:**
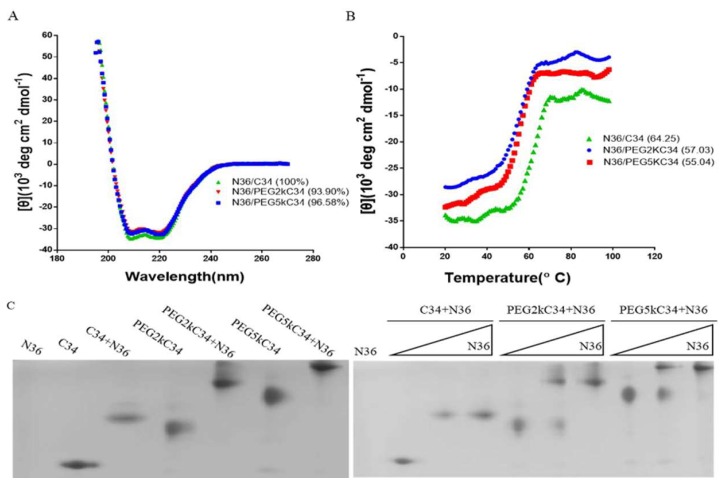
Analysis of the affinity of N36 and the designed peptides by CD spectroscopy and N-PAGE. (**A**) The α-helical content was calculated from the CD signal by dividing the mean residue ellipticity at 222 nm. (**B**) The midpoint of the thermal unfolding transition of the peptides. (**C**) N-PAGE analysis of 6-HB formation between N36 and C34 as well as PEGylated C34 peptides. The final concentration of the peptides N36, C34, PEG2kC34 and PEG5kC34 was 100 μM. The gradient concentration of the peptides N36 on the right Figure 2C was 0, 50 and 100 μM, respectively.

We explored the affinity between the PEGylated C34 and the NHR peptide N36, which contains the full binding region for C34, using a model suitable for the investigation of HIV fusion inhibitors [23]. CD spectroscopy detected the specific interaction between the NHR peptide N36 and C34, as well as the PEGylated C34 peptides. Like the N36/C34 complex, the N36/PEGylated C34 complexes exhibited high α-helicity content (Figure 2A), although the melting temperature, defined as the midpoint of thermal unfolding transition, of the N36/PEGylated C34 complexes was slightly lower than that of N36/C34 complex (Figure 2B). N-PAGE analysis showed that like C34, both PEGylated C34 peptides could combine with N36 to form 6-HB bands at the higher position than the bands of individual C34 or PEGylated C34. The 6-HB bands has a dose-dependent relationship with N36 in a certain range (Figure 2C). N36 showed no band in N-PAGE because it carries net positive charge, making the peptide migrate up and off the gel [24].

### 3.3. Inhibitory Potency of PEGylated C34 against Env-Mediated Cell–Cell Fusion and Infection of Laboratory-Adapted HIV-1 Strain with Low Cytotoxicity

**Figure 3:**
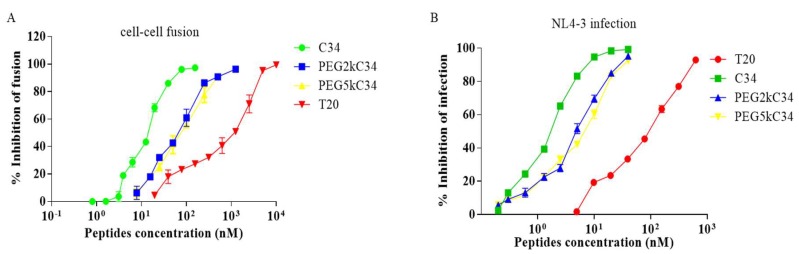
The inhibitory activities of the peptides. (**A**) The inhibitory activities of T20, C34, PEG2kC34 and PEG5kC34 against cell–cell fusion. (**B**) The infection inhibitory activities of T20, C34, PEG2kC34 and PEG5kC34 against the laboratory-adapted strain NL4-3 in TZM-bl cells.

The inhibitory activity of PEGylated C34 against HIV-1 Env-mediated cell–cell fusion was evaluated using CD4 and coreceptor-expressing TZM-bl cells as target cells and HIV-1 Env-expressing HEK293T cells as effector cells. The peptides PEG2kC34 and PEG5kC34 could effectively inhibit cell–cell fusion with an EC_50_ of about 36 nM (Figure 3A). They also inhibited replication of the laboratory-adapted HIV-1 strain NL4-3 with EC_50_ of about 4–5 nM (Figure 3B). The cytotoxicity of PEGylated C34 was evaluated in TZM-bl cells. As expected, the peptide exhibited low cytotoxicity on TZM-bl cells with CC_50_ values greater than 100 μM.

### 3.4. PEGylated C34 Could Effectively Inhibit the Replication of the HIV-1 Subtypes Circulating in China

**Figure 4:**
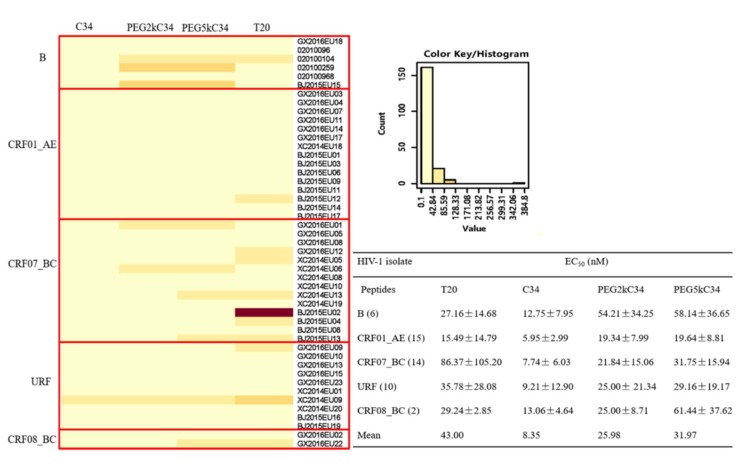
The infection inhibitory activities of T20, C34, PEG2kC34 and PEG5kC34 against 47 clinical isolates in TZM-bl cells. The 47 clinical isolates are B (6/47), CRF01_AE (15/47), CRF07_BC (14/47), URF (10/47) and CRF_08BC (2/47) subtypes. The heat map was constructed with the online software (HIV database). Low values tend towards yellow tones while higher values tend to hotter orange and red tones.

Some reports suggested that HIV subtypes might affect their susceptibility to certain antiretroviral drugs, which could influence therapeutic outcomes [25]. Therefore, in this study, we tested the inhibitory activities of the peptides on 47 strains of HIV-1 isolates circulating in China (Tables S1 and S2). The results showed that PEGylated C34 peptides had lower EC_50_ on HIV-1 isolates circulating in China, when compared to T20, especially for the CRF07_BC subtype (Figure 4), which has been predominant in injection drug users [26]. HIV-1 subtype B isolates had the highest EC_50_ to PEGylated C34, while CRF01_AE subtypes had the lowest EC_50_ to PEGylated C34 in this study (Figure 4). However, sensitivity results for subtype CRF08_BC need to be further confirmed owing to the small sample size. Recombination between HIV-1 subtypes is one of the main mechanisms leading to the genetic diversity and complexity of the HIV-1 genome. This study analyzed the drug susceptibility of 10 unique recombinant forms (URFs). Their EC_50_ was basically consistent with the mean of the EC_50_. 

### 3.5. Decreased Resistance of T20-Induced HIV-1 Mutants to PEGylated HIV-1 Fusion Inhibitors

To identify the inhibitory activities of PEGylated peptide against known T20 resistant viral strains, we generated several HIV-1 NL4-3 mutants carrying the amino acid substitutions either singly or in combination. The corresponding infectious virus was then produced and used in infection assay. The results showed that the viruses that contained the V38A, N43K, V38A/N42D or V38A/N42T were resistant to T20 (3–11 fold), C34 (15–156 fold), PEG2kC34 (7–28 fold) and PEG5kC34 (7–16 fold). The resistant fold of PEG2kC34 (7–28 fold) and PEG5kC34 (7–16 fold) decreased compared to C34 (15–156 fold) (Table 1).

### 3.6. PEGylated C34 Displayed Prolonged Half-Life

**Figure 5:**
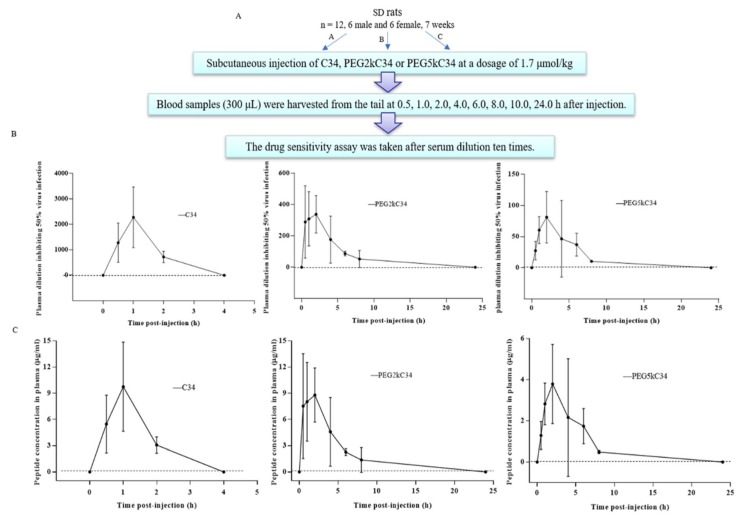
The ex vivo anti-HIV-1 activity of the peptides tested. (**A**) Three groups of SD rats (2 male and 2 female rats/group) were subcutaneously administered with peptides C34, PEG2kC34 and PEG5kC34, respectively, and rat plasma were harvested before injection and at different time points (0.5, 1.0, 2.0, 4.0, 6.0, 8.0, 10.0 and 24.0 h) after injection. (**B**) Antiviral activities of C34, PEG2kC34 and PEG5kC34 in rat plasma. Plasma dilutions required for 50% inhibition of HIV-1 strain NL4-3infection were determined. (**C**) Concentration of the active peptides in the plasma samples was estimated. Error bars represent the standard deviation of each mean.

The protocol for determining the pharmacokinetics of PEGylated C34 in rats is shown. (Figure 5A). The HIV-1 inhibitory activity of plasma from rats treated with C34 reached the highest peak level at 1 h, then dropped rapidly, and decreased to an undetectable level at 4 h post injection, with a plasma half-life (t_1/2_) of 1.1 h. The plasma from rats treated with PEG2kC34 and PEG5kC34 reached the highest peak level at 2 h then dropped slowly and decreased to an undetectable level at 24 h post injection (Figure 5B). Based on the in vitro EC_50_ and ex vivo anti-HIV-1 activity of PEGylated C34 for inhibiting NL4-3 infection, we estimated the concentration of the active peptides in the plasma of rats collected at different times (Figure 5C). Then, we calculated the pharmacokinetic parameters of them (Table 2). The plasma t_1/2_ of PEG2kC34 and PEG5kC34 was 2.6 and 5.1 h, respectively, and the t_1/2_ of PEG5kC34 was about 3.4-fold and 4.6-fold longer than T20 (~1.5 h) [12] and C34 (~1.1 h). These results suggest that PEG modification of C34 results in a significant decreased rate of renal filtration of the peptide and prolonged half-life of the peptide-based HIV fusion inhibitor. 

## 4. Discussion

Anti-HIV drugs play an important role in preventing and treating AIDS [27], a series of ARTs, including nucleoside reverse transcriptase inhibitors (NRTIs), non-nucleoside reverse transcriptase inhibitors (NNRTIs), integrase inhibitors (IIs), protease inhibitors (PIs), fusion/entry inhibitors (EIs), and pharmacokinetic enhancers, have been developed [28]. ART has successfully transformed HIV-1 infection into a chronic and manageable disease [29]. However, prevalence of drug resistance and treatment is only effective before HIV-1 develops resistance against the administered drugs [30]. HIV fusion inhibitors have received widespread attention as they act extracellularly, prior to invasion of the host cell [31]. The first approved HIV fusion inhibitor by the U.S. FDA, T20 was used to treat HIV-infected patients who failed to respond to the current antiretroviral drugs [23]. But its clinical application was limited mainly due to its half-life [32]. This calls for the development of new HIV fusion drugs. Many approaches have been developed to improve the pharmaceutical profiles of HIV-1 fusion inhibitors. Peptide engineering strategies, such as the incorporation of salt bridges [33,34], and chemical modification, such as lipid [34], albumin [35,36], glycan [14], or cholesterol [22], have been applied to increase the stability and antiviral activity of peptide sequences or reduce immunogenicity and proteolysis [37,38]. PEGylation is able to prolong the half-life of a drug by reducing its clearance through glomerular filtration, the reticuloendothelial system or proteolytic degradation [11]. Huet [39] and Cheng [12] applied PEG to improve the pharmacokinetic properties of the first HIV fusion inhibitor-Enfuvirtide. Compared with T20, C34 is a more potent inhibitor [40]. It has been widely used as a design template [10]. Quinn [41] designed and assessed pre-clinical toxicology and performed the first in-human administration of C34-PEG_4_-Chol in their study, but there is no action mechanism involved. In this study, we employed this convenient and economical strategy to improve the pharmacokinetics of C34 and explored their action mechanisms. This method can solve some bottleneck problems in chemical modification, such as the multi-point random modification, which result in reduced product activity, complex composition, low efficacy or severe reaction conditions, causing conformational change. Two conjugates, PEG2KC34 and PEG5kC34 were successfully synthesized. The conjugates showed broad spectrum anti-HIV-1 activity with low cytotoxicity. They exhibited potent anti-fusion activity and anti-viral activity for most isolates. The sensitivities of HIV-1 to different drugs might be affected by mutation sites, subtypes and co-receptors. Earlier studies to measure the HIV mutation rate in vivo had determined a forward mutation rate of 3 × 10^−5^ mutations per target base pair, per replication cycle [29]. The high mutation rate by HIV generates a genetically diverse set of viruses in the presence or absence of drugs, affecting the sensitivity of HIV-1. In this study, we analyzed the drug susceptibility of 47 HIV-1 clinical isolates from Beijing, Guangxi, Sichuan and Anhui to PEGylated C34. Compared with other subtypes in this study, the CRF01_AE subtype is more sensitive to HIV-1 fusion inhibitors. The CRF01_AE subtype is the major epidemic HIV-1 with clusters circulating in mainland China [42]. Thus HIV-1 fusion inhibitors may be the promising candidate for treating and preventing HIV-1 infection. It is reported that in the early stage of HIV-1 infection, the co-receptor of HIV-1 is CCR5, which is then gradually converted into CCR5/CXCR4, and finally, further converted to CXCR4 [43]. Because 47 strains of the virus in this study were mainly CCR5 strains, only 4 strains of CCR5/CXCR4, and no CXCR4 strain was detected, the effect of HIV-1 co-receptor utilization on the antiviral activity of HIV-1 fusion inhibitors could not be further analyzed. 

PEGylation of HIV fusion inhibitors have been described in numerous studies prior, such as PCT patents WO2004013164 and WO2004013165. But they chose to analyze the larger molecular weight PEG 10 kDa or PEG 20 kDa. As reported, the position and chain length of PEG conjugation can affect the HIV-1 membrane fusion inhibition and proteolytic degradation [44]. The addition of a group to the C-terminal of an HIV-1 peptide fusion inhibitor decreased its antiviral potency. And the bulky polymer shells might have given rise to transport limitations to- and from the viral target that exert an overall negative impact on antiviral potency [44]. In this study, we chose to modify C34 with smaller PEG2kD and PEG5kD at its N-terminus, trying to find the judicious choice of the position and chain length of conjugation which enables access to PEGylated HR2 with only slightly reduced fusion inhibitory efficacies, as compared to the non-PEGylated peptide, but with significantly improved proteolytic stabilities [45]. Circular dichroism spectroscopy results showed that both PEGylated fusion inhibitors could bind to the corresponding sequences of HIV-1 gp41 to form high α-helical complexes. The α-helix or melting temperatures of the composites N36/PEG2kC34 and N36/PEG5kC34 were 93.90% and 96.58% or 57.03 °C and 55.04 °C, respectively. This might one explanation for the decreased antiviral activity of PEGylated C34, compared to before modification. Our study indicated that PEGylation could significantly extend half-life, which was consistent with previous studies [12]; but, different from the studies in which it was shown that the immune system may produce specific PEG antibodies that result in accelerated blood clearance of PEGylated therapeutics [46]. In the early stage, we cooperated with the Institute of Microbiology of the Chinese Academy of Sciences to identify the plasma half-life of ENF and PEG2k-ENF by HPLC. The results showed that the half-life of PEG2k-ENF was prolonged by 10-fold. Due to the plasma C34 UV absorption peak is not easily distinguished from other plasma components. We chose an indirect method, as described by Jiang and colleagues [20], to detect the plasma half-life (t_1/2_) of PEGylated C34 peptides in this study. We detected the dilutions of plasma at different time points that inhibited 50% viral replication, based on which the plasma half-life (t_1/2_) of PEGylated C34 peptides in SD rats was calculated. We found that the plasma t_1/2_ of PEG2kC34 and PEG5kC34 was prolonged, suggesting that the PEGylated C34 had better potential for further development, which would be a better choice for a HIV-1 fusion inhibitor candidate for further study. 

## 5. Conclusions

We designed and synthesized two peptides, PEG2kC34 and PEG5kC34, and demonstrated that they not only exhibited potent antiretroviral activity against 47 HIV-1 clinical isolates circulating in China, but also had a long half-life.

## Figures and Tables

**Table 1:** Resistance of T20-Induced HIV-1 Mutants to PEGylated HIV-1 Fusion Inhibitors.

HIV-1 Strains	T20		C34		PEG2kC34		PEG5kC34	
	EC_50_	n-Fold	EC_50_	n-Fold	EC_50_	n-Fold	EC_50_	n-Fold
NL4-3	128.53 ± 21.06	1	0.98 ± 1.12	1	4.14 ± 1.89	1	4.59 ± 1.83	1
NL4-3_V38A_	1361.21 ± 72.93	10.6	14.44 ± 5.44	14.7	34.10 ± 18.07	8.2	31.49 ± 4.71	6.9
NL4-3_N43K_	764.40 ± 398.35	5.9	15.24 ± 5.73	15.6	30.03 ± 10.29	7.3	39.33 ± 32.10	8.6
NL4-3_V38A/N42D_	424.70 ± 290.16	3.3	26.07 ± 4.77	26.6	59.39 ± 39.03	14.4	45.91 ± 38.62	10.0
NL4-3_V38A/N42T_	780.06 ± 426.99	6.07	153.13 ± 19.80	156.3	114.71 ± 36.06	27.7	71.12 ± 38.07	15.5

**Table 2:** Pharmacokinetic parameters of PEG2kC34 and PEG5kC34 in rats.

Parameter	PEG2kC34	PEG5kC34
	Rat, i.h., *n* = 4	
T_max_ (h)	2.94 ± 1.30	3.88 ± 1.77
C_max_ (μg/mL)	10.37 ± 3.55	4.32 ± 1.88
t_1/2_ (h)	2.57 ± 0.71	5.11 ± 3.54
AUC_0-24h_ (μg/mL * h)	39.75 ± 16.80	17.68 ± 6.98
AUCINF_obs (μg/mL * h)	40.38 ± 16.75	15.56 ± 2.63
Vz_F_obs (ml/kg)	773.42 ± 355.01	2688.46 ± 341.05
Cl_F_obs (ml/h/kg)	353.43 ± 84.54	1033.79 ± 163.89
MRTlast (h)	3.08 ± 0.55	3.75 ± 0.44

## Supplementary Materials

The following are available online at https://www.mdpi.com/1999-4915/11/9/811/s1, Table S1: The detail information about the EC_50_ of PEGylated peptide against each virus. Table S2: The detail information about the HIV gp41 sequence.
