# Supplementary material for: Long-Acting HIV-1 Fusion Inhibitory Peptides and their Mechanisms of Action
Source: Viruses. 2019 Sep 2;11(9):811. doi: 10.3390/v11090811 (PMC6784077; doi:10.3390/v11090811)
Supplement: Supplementary file 1 [file viruses-11-00811-s001.zip › viruses-573027-supplementary/Supplement 1.docx]

Table 1 Sensitivity of CRF01_AE subtype HIV-1 isolates to HIV-1 fusion inhibitors (15 strains)

|  | EC_50_(nM) | | | |
| --- | --- | --- | --- | --- |
| HIV-1 isolates | T20 | C34 | PEG2kC34 | PEG5kC34 |
| GX2016EU03 | 17.66 ± 11.75 | 10.25 ± 5.50 | 31.63 ± 17.67 | 30.28 ± 15.65 |
| GX2016EU04 | 27.37 ± 10.35 | 6.89 ± 3.85 | 27.87 ± 12.23 | 21.33 ± 8.93 |
| GX2016EU07 | 11.57 ± 8.13 | 2.30 ± 1.73 | 23.39 ± 10.77 | 13.15 ± 10.36 |
| GX2016EU11 | 19.46 ± 5.98 | 5.81 ± 2.37 | 24.16 ± 11.02 | 24.73 ± 10.03 |
| GX2016EU14 | 8.97 ± 6.45 | 10.79 ± 4.33 | 24.42 ± 13.53 | 26.27 ± 17.55 |
| GX2016EU17 | 9.36 ± 7.89 | 6.49 ± 4.61 | 12.76 ± 9.74 | 16.53 ± 11.76 |
| XC2014EU18 | 10.00 ± 7.75 | 4.62 ± 3.64 | 9.65 ± 6.72 | 14.64 ± 9.47 |
| BJ2015EU01 | 1.00 ± 0.49 | 6.81 ± 2.59 | 29.42 ± 15.12 | 40.05 ± 13.36 |
| BJ2015EU03 | 35.64 ± 24.71 | 6.23 ± 3.52 | 13.25 ± 9.65 | 10.92 ± 7.25 |
| BJ2015EU06 | 9.32 ± 7.14 | 3.73 ± 3.20 | 18.47 ± 12.91 | 14.85 ± 11.59 |
| BJ2015EU09 | 9.99 ± 8.11 | 10.84 ± 4.54 | 22.72 ± 11.54 | 22.35 ± 12.59 |
| BJ2015EU11 | 0.90 ± 0.35 | 1.52 ± 0.60 | 7.46 ± 4.56 | 11.30 ± 2.13 |
| BJ2015EU12 | 58.80 ± 47.59 | 6.79 ± 3.44 | 12.13 ± 7.53 | 12.58 ± 7.78 |
| BJ2015EU14 | 11.05 ± 9.49 | 0.87 ± 0.73 | 7.14 ± 5.15 | 6.60 ± 4.98 |
| BJ2015EU17 | 1.33 ± 1.35 | 5.28 ± 3.84 | 25.58 ± 17.06 | 29.08 ± 16.60 |
| Mean EC_50_ | 15.49 ± 14.79 | 5.95 ± 2.99 | 19.34 ± 7.99 | 19.64 ± 8.81 |

Table 2 Sensitivity of CRF07_BC subtype HIV-1 isolates to HIV-1 fusion inhibitors (14 strains）

| HIV-1 isolates | EC_50_(nM) | | | |
| --- | --- | --- | --- | --- |
|  | T20 | C34 | PEG2kC34 | PEG5kC34 |
| GX2016EU01 | 26.20 ± 114.07 | 13.07 ± 3.45 | 52.94 ± 10.18 | 48.62 ± 8.77 |
| GX2016EU05 | 311.59 ± 81.91 | 15.94 ± 3.98 | 18.93 ± 5.76 | 32.85 ± 7.87 |
| GX2016EU08 | 36.63 ± 26.65 | 0.22 ± 0.21 | 10.15 ± 6.25 | 30.97 ± 11.53 |
| GX2016EU12 | 56.32 ± 38.85 | 4.60 ± 2.43 | 10.25 ± 6.96 | 35.99 ± 14.87 |
| XC2014EU05 | 72.07 ± 6.81 | 14.46 ± 1.02 | 31.51 ± 5.75 | 37.63 ± 1.00 |
| XC2014EU06 | 33.29 ± 20.94 | 10.25 ± 3.78 | 46.72 ± 8.66 | 45.93 ± 19.63 |
| XC2014EU08 | 37.98 ± 15.88 | 19.97 ± 9.34 | 34.47 ± 12.50 | 25.97 ± 18.16 |
| XC2014EU10 | 30.19 ± 23.17 | 3.16 ± 1.93 | 4.56 ± 0.74 | 8.70 ± 6.65 |
| XC2014EU13 | 44.34 ± 27.23 | 0.58 ± 0.32 | 9.53 ± 6.93 | 46.73 ± 11.61 |
| XC2014EU19 | 15.49 ± 8.21 | 0.29 ± 0.22 | 0.83 ± 0.63 | 4.45 ± 1.46 |
| BJ2015EU02 | 384.79 ± 92.14 | 4.86 ± 3.77 | 13.43 ± 8.29 | 15.27 ± 9.75 |
| BJ2015EU04 | 74.53 ± 28.92 | 11.50 ± 3.17 | 28.58 ± 10.99 | 32.04 ± 13.05 |
| BJ2015EU08 | 42.11 ± 34.92 | 2.12 ± 1.42 | 10.21 ± 8.18 | 13.82 ± 2.12 |
| BJ2015EU13 | 43.66 ± 21.43 | 7.39 ± 6.65 | 33.61 ± 12.72 | 65.59 ± 5.87 |
| Mean EC_50_ | 86.37 ± 105.20 | 7.74 ± 6.03 | 21.84 ± 15.06 | 31.75 ± 15.94 |

Table 3 Sensitivity of CRF08_BC subtype HIV-1 isolates to HIV-1 fusion inhibitors (2 strains）

| HIV-1 isolates | EC_50_(nM) | | | |
| --- | --- | --- | --- | --- |
|  | T20 | C34 | PEG2kC34 | PEG5kC34 |
| GX2016EU02 | 26.39 ± 18.67 | 8.41 ± 3.94 | 16.28 ± 8.30 | 23.82 ± 13.93 |
| GX2016EU22 | 32.08 ± 25.53 | 17.70 ± 8.76 | 33.71 ± 17.04 | 99.05 ± 39.04 |
| Mean EC_50_ | 29.24 ± 2.85 | 13.06 ± 4.64 | 25.00 ± 8.71 | 61.44 ± 37.62 |

Table 4 Sensitivity of B subtype HIV-1 isolates to HIV-1 fusion inhibitors (6 strains)

| HIV-1 isolates | EC_50_(nM) | | | |
| --- | --- | --- | --- | --- |
|  | T20 | C34 | PEG2kC34 | PEG5kC34 |
| GX2016EU18 | 8.51 ± 7.39 | 8.50 ± 4.49 | 24.50 ± 17.00 | 22.27 ± 10.97 |
| 02010096 | 16.53 ± 4.25 | 7. 18 ± 2.93 | 22.95 ± 15.34 | 23.79 ± 13.46 |
| 02010104 | 50.69 ± 27.68 | 10.00 ± 3.43 | 57.91 ± 16.23 | 76.06 ± 4.53 |
| 020100259 | 29.60 ± 19.65 | 22.46 ± 11.11 | 93.04 ± 42.36 | 92.07 ± 38.89 |
| 020100968 | 17.27 ± 14.80 | 4.21 ± 2.41 | 21.64 ± 9.04 | 22.88 ± 11.92 |
| BJ2015EU15 | 40.36 ± 15.00 | 24.14 ± 3.82 | 105.19 ± 8.40 | 111.79 ± 23.75 |
| Mean EC_50_ | 27.16 ± 14.68 | 12.75 ± 7.95 | 54.21 ± 34.25 | 58.14 ± 36.65 |

Table 5 Sensitivity of HIV-1 recombinant isolates to HIV-1 fusion inhibitors (10 strains)

| HIV-1 isolates | EC_50_(nM) | | | |
| --- | --- | --- | --- | --- |
|  | T20 | C34 | PEG2kC34 | PEG5kC34 |
| GX2016EU09 | 49.54 ± 24.54 | 1.28 ± 1.09 | 26.97 ± 12.46 | 32.92 ± 10.65 |
| GX2016EU10 | 27.50 ± 16.22 | 0.39 ± 0.34 | 15.38 ± 5.73 | 20.35 ± 10.90 |
| GX2016EU13 | 8.78 ± 7.44 | 9.69 ± 7.31 | 30.41 ± 20.08 | 27.03 ± 11.01 |
| GX2016EU15 | 32.40 ± 15.07 | 13.90 ± 5.60 | 22.71 ± 10.88 | 36.95 ± 14.89 |
| GX2016EU23 | 4.78 ± 4.08 | 0.11 ± 0.092 | 0.93 ± 0.82 | 2.26 ± 1.49 |
| XC2014EU01 | 39.19 ± 26.54 | 4.95 ± 3.68 | 23.71 ± 15.48 | 27.32 ± 15.84 |
| XC2014EU09 | 110.33 ± 21.89 | 45.85 ± 5.94 | 84.22 ± 10.63 | 78.39 ± 5.43 |
| XC2014EU20 | 25.48 ± 17.22 | 2.76 ± 1.35 | 10.25 ± 5.00 | 11.59 ± 3.24 |
| BJ2015EU16 | 39.50 ± 31.47 | 7.55 ± 4.93 | 20.32 ± 9.34 | 22.16 ± 15.20 |
| BJ2015EU19 | 20.25 ± 12.43 | 5.58 ± 2.67 | 15.14 ± 6.92 | 32.63 ± 3.98 |
| Mean EC_50_ | 35.78 ± 28.08 | 9.21 ± 12.90 | 25.00 ± 21.34 | 29.16±19.17 |
