# Supplementary material for: Long-Acting HIV-1 Fusion Inhibitory Peptides and their Mechanisms of Action
Source: Viruses. 2019 Sep 2;11(9):811. doi: 10.3390/v11090811 (PMC6784077; doi:10.3390/v11090811)
Supplement: Supplementary file 1 [file viruses-11-00811-s001.zip › viruses-573027-supplementary/Supplement 2.docx]

>020100096

GCAGTGGGAATAGGAGCTATGTTTCTTGGGTTCTTGGGAGCAGCAGGAAGCACTATGGGCGCAGCGTCAATAACGCTGACGGTACAGGCCAGACAATTGTTGTCTGGTATAGTGCAACAGCAGAGAAATCTGCTGAGGGCTATTGAGGCGCAACAGCATCTGTTGCAACTCACAGTCTGGGGCATCAAGCAGCTCGTGGCAAGAGTCTTGGCTGTGGAAAGATACCTAAAGGATCAACAGCTCCTAGGGATTTGGGGTTGCTCTGGAAAACTCATCTGCACCACTGCTGTGCCTTGGAATGTTAGTTGGAGTAATAAATCCCTGGATGAGATTTGGAATAACATGACTTGGATGGAGTGGGAAAGAGAAATAGACAATTACACAAGAGAAATATATACCTTAATTGAAGAATCGCAGAACCAACAAGAGAAGAATGAGCTAGAACTATTGGAATTGGATAAGTGGGCAAGTTTGTGGAATTGGTTTGACATAACAAAATGGCTGTGGTATATCAAAATATTCATAATGATAGTAGGGGGCTTGGTAGGTTTAAGAATAGTTTTTGCTGTACTTTCTATAGTAAATAGAGTTAGGCAGGGATACTCACCATTGTCATTACAGACCCGCTTCCCAACCCAGAGGGGACCCGGCAGGCCCGAAGGAATCGAAGAAGAAGGTGGAGAGCAAGACAGAGACAGATCCGAGAGATTAGTGAACGGATTCTTGACACTTTTCTGGGAGGATCTACGGAACCTGTGCCGCTTCAGTTACCACCGATTGAGAGACTTACTCTTGATTGTAGCAAGGATTGTGGAACTTCTGGGACGCA---------------------GGGGGTGGGAAGCCCTCAGATATTGGTGGAATCTCCTGCAGTATTGGATTCAGGAACTAAAGAATAGTGCTATTAGCTTGCTTAACGCCACAGCCATAGCAGTAACTGAGGGGACAAATAGGGTCATAGAAGTAGCACAAAGAGCTTATAGGGCTATTCTCAACATACCTACAAGAATCAGACAGGGCCTCGAAAGGGCTTTGCTGTAA

>020100104

GCAGTGGGAATAGGAGCTATGTTCCTTGGGTTCTTGGGAGCAGCAGGAAGCACTATGGGCGCAGCGTCAATAACGCTGACGGTACAGGCCAGACAATTGTTGTCTGGTATAGTGCAGCAGCAGGGAAATCTGCTGAGGGCTATTGAGGCGCAACAGCATCTGTTGCAACTCACAGTCTGGGGCATCAAGCAGCTCCAGGCAAGAGTCTTGGCTGTGGAAAGATACCTAAAGGATCAACAGCTCCTAGGGCTTTGGGGTTGCTCTGGAAAACTCATTTGCACCACTAATGTGCCTTGGAATACTAGTTGGAGTAATAGATCTCTGAGTGAGATTTGGGATAACATGACTTGGATGGAATGGGAAAGAGAGATTAACAATTACACAAGAGTAATATACACCTTAATTGAAGAATCGCAAAACCAACAAGAAAAGAATGAACTAGAACTATTGGAATTGGATAAGTGGGCAAGCTTGTGGAATTGGTTTGACATAACAAATTGGCTGTGGTATATCAAAATATTCATAATGATAGTAGGAGGCTTGGTAGGTTTAAGAATAGTTTTTGCTGTACTTTCTATAGTAAATAGAGTTAGGCAGGGATACTCACCATTGTCATTACAGACCCGCTTCCCAACCCAGAGGGGACCCGACAGGCCCGAAGGAATCGAAGAAGAAGGTGGAGAGCGAGACAGAGACAGATCCGAGAGATTAGTGAACGGATTCTTGACACTTATCTGGGAGGATCTACGGAACCTGTGCCTCTTCAGCTACCACCGCTTGAGAGACTTACTCTTGATTGTAGCCAGGATTGTGGAACTTCTGGGACGCA---------------------GGGGGTGGGAAGCCCTCAGATATTGGTGGAATCTCCTGCAGTATTGGATTCAGGAACTAAAGAATAGTGCTATTAGCTTGTTTAACGCCACAGCCATAGCAGTAGCTGAAGGGACAGATAGGGTTATAGAAATAGTACAAAGAGCTTATAGGGCTATTCTCAACATACCTACAAGAATCAGACAGGGCCTCGAAAGGGCTTTGCTATAA

>020100259

GCAGTGGGAATAGGAGCTATGTTCCTTGGGTTCTTGGGAGCAGCAGGAAGCACTATGGGCGCAGCGTCAATGACGCTGACGGTACAGGCCAGACAATTGTTGTCTGGTATAGTGCAACAGCAGAGAAATCTGCTGAGGGCTATTGAGGCGCAACAGCATCTGTTGCAACTCACAGTCTGGGGCATCAAGCAGCTCCAGGCAAGAGTCTTGGCTGTGGAAAGATACCTAGGCGATCAACAGCTCCTAGGGATTTGGGGTTGCTCTGGAAAACTCATCTGCACCACTGCTGTGCCTTGGAATGTTAGTTGGAGTAATAAGTCTCATGATGAGATTTGGAATAACATGACTTGGATGGAGTGGGAAAGAGAAATTGAAAATTACACAAAAGAAATATACACTTTACTTGGAGAATCGCAGAACCAACAAGAAAAGAATGAGCAAGAACTATTGGAATTGGATAAGTGGGCAAGCTTGTGGAACTGGTTTAACATATCAAATTGGCTGTGGTATATCAAAATATTTATAATGATAGTAGGAGGCTTGGTAGGTTTAAGAATAGTTTTTGCTGTACTTTCTATAGTAAATAGAGTTAGGCAGGGATACTCACCATTGTCATTACAGACCCGCCTCCCAACCCAGAGGGGACCCGGCAGGCCCGAAGGAATCGAAGAAGAAGGTGGAGAGCAAGACAGAGACAGATCCGAGAGATTAGTGAACGGATTCTTGCCACTTATCTGGGAGGATCTACGGAGCCTGTGCCTCTTCAGCTACCACCTCTTGAGAGACTTACTCTTGATTGTAGCAAGGACTGTGGAACTTCTGGGACGCA---------------------GGGGGTGGGAAGCCCTCAGATATTGGTGGAATCTCCTGCAGTATTGGATTCAGGAACTAAAGAATAGTGCTATTAGCTTGCTTAACGCTACAGCCATAGCAGTAGCTGAGGGGACAGACAGGGTTATAGAAATAGCACAAAGAGCTTTTAGGGCTATTCTCAACATACCTAGAAGAATCAGACAGGGCCTCGAAAGGGCTTTGCTATAA

>020100968

GCAGTGGGAATAGGAGCTATGTTCCTTGGGTTCTTGGGAGCAGCAGGAAGCACTATGGGCGCAGCGTCAATAACGCTGACGGTACAGGCCAGACAATTGTTGTCTGGTATAGTGCAACAGCAGAGAAATTTGCTGAGGGCTATTGAGGCGCAACAGCATCTGTTGCAACTCACAGTCTGGGGCATCAAACAGCTCCAGGCAAGAGTCTTGGCTGTGGAAAGATACCTACAGGATCAACAGCTCCTAGGGATTTGGGGTTGCTCTGGAAAACTCATTTGCACCACTGCTGTGCCTTGGAATGTTAGTTGGAGTAATAAATCTCTGAGTGAGATTTGGGATAACATGACTTGGATGGAGTGGGAAAGAGAAATTGGCAATTACACAAGTCAAATATACACCTTAATTGAACAATCGCAGAACCAACAAGAAAAGAATGAACTAGAACTATTGGAATTGGATAAATGGGCAAGTTTGTGGAATTGGTTTAGCATAACAAACTGGCTGTGGTATATCAAAATATTCATAATGATAGTAGGAGGCTTGGTAGGTTTAAGAATAATTTTTGCTGTACTTTCTGTAGTAAATAGAGTTAGGCAGGGATACTCACCTTTGTCATTACAGACCCGCTTCCCAACCCAGAGGGGACCCGGAAGGCCCGAAGGAATCGAAGAAGAAGGTGGAGAGCGAGACAGAGACAGATCCGAGAGATTAGTGAACGGATTCTTGACACTTTTCTGGGAGGATCTAAGGAGCCTGTGCCTCTTCAGCTACCACCGCTTGAGAGACTTACTCTTGATTGTAGCAAGGATTGTGGAACTTCTGGGACGCA---------------------GGGGGTGGGAAGTCCTCAGATATTGGTGGAATCTCCTGCAGTATTGGATACAGGAACTAAAGAATAGTGCTGTTAGCTTGCTTAACGCCACAGCCATAGCAGTAGCTGAGGGGACAGATAGGATTATAGAATTAGCACAAAGAGCTTTTAGGGCTTTTCTCAACATACCTACAAGAATCAGACAGGGCCTCGAAAGGGCTTTGCTATAA

>GX2016EU01

GCAGTGGGAATAGGAGCTGTGTTCCTTGGGTTCTTGGGAGCAGCAGGAAGCACTATGGGCGCGGCGTCAATAACGCTGACGGTACAGGCCAGACTATTGCTGTCTGGTATAGTGCAACAGCAAAGCAATTTGCTGAGAGCTATAGAGGCGCAACAGCATCTGTTGCAACTCACAGTCTGGGGCATTAAGCAGCTCCAGACAAGAATCCTGGCTATAGAAAGATACCTAAAGGATCAGCAGCTCCTAGGGATTTGGGGCTGCTCTGGAAAACTCATCTGCACTACTGCTGTACCTTGGAACTCCAGTTGGAGTAGCAAGACTAAAGACGAGATTTGGAATAACTTGACCTGGATGGAATGGGATAGAGAAATTAGCAATTACACAGACATAATATACCAGTTGCTTGAAGAATCGCAAAACCAGCAGGAAAGGAATGAAAAGGATCTATTAGCATTGGACAGTTGGAATAGTCTATGGAATTGGTTCAACATAACAAGTTGGCTGTGGTATATAAAAATATTCATAATAATAGTAGGAGGCCTGATAGGTTTAAGAATATTTTTTGCTGTGCTTTCTATAGTGAATAGAGTTAGGCAGGGATACTCACCTTTGTCATTTCAGATCCTTACCCCGAACCCAGGGGGACCCGACAGGCTCGGAAGAATCGAAGAAGAAGGTGGAGAGCAAGACAGAGAGAGATCCATTCGCTTAGTGAACGGATTCTTAGCACTTGCCTGGGACGACCTGCGGAACCTGTGCCTCTTCAGCTACCACCGCTTGAGAGACTTCATATTAGTGGCAGCGAGAGTGGTGGAACTYCTGGGAC---------------------AGAGGGGGTGGGAAACCCTTAAATATCTGGGAAGTCTTGGGCAGTACTGGGGTCAGGAGCTAAAAAAGAGCGCTATCAGTCTGTTTGATACCATAGCAATAGCAGTAGCTGAAGGAACAGATAGGGTTATAGAATTAATACGAGGATTTTGTAGAGCTATCTACAACATACCTAGAAGAATAAGACAGGGCCTTGAAGCAGCTTTGCAATAA

>GX2016EU02

ACAGCA---CTAGGAGCTGTGTTCCTTGGGTTCTTGGGATTAGCCGGAAGCACTATGGGCGTGGCGTCATTGACGCTGACGGTACAGGCCAGACAATTGTTGTCTGGTATAGTGCAACAGCAAAGCAATTTGCTGAGAGCTATAGAGGCGCAACAGCATATGTTGCAACTCACGGTCTGGGGCATTAAGCAGCTACAGACAAGAGTCCTGGCTATAGAAAGATACCTAAGGGATCARCAGCTCCTAGGAATTTGGGGCTGCTCTGGAAAACTTATCTGCACTACTAATGTRCCTTGGAACTCCAGTTGGAGTAACAAATCTCAMCAAGAGATTTGGRASAACATGACCTGGATGCAGTGGGATAAGGAAATTAATAATTACACARRCAYAATATACAGGTTGCTTGAAGAATCGCAAAACCAGCAGGAAAACAATGAAAAAGATTTATTAGCATTGGACAGTTGGAACACTCTATGGAGTTGGTTTAACATATCAAATTGGCTGTGGTATATAAGAATATTCATAATGATAGTAGGAGGCTTGATAGGTTTAAGAATAATTTTTGCTGTGCTTTCTATAGTGAATAGAGTTAGGCAGGGATACTCACCTTTGTCGTTCCAGATCCCTACCCCGAACCCAGGGGGACCAGACAGGCTCRGAGGAATCGAAGAAGAAGGTGGAGAGCAAGACAAAACCAGATCCATTCGATTAGTGAACGGGTTCTTAGCRCTTGCCTGGGAAGACCTGCGGAACCTGTGCCTCTTCAGTTACCACCGCTTGAGAGACTTAATATTACTGACAGCGAGGGGAGTGGAACTTCTGGGACGCAACAGCCTCAAGGGAATACAGAGGGGGTGGGAAGCCCTTAAATACCTGGGAAGCCTTGTGCAGTATTGGGGTYTAGAGCTAAAAAAGAGTACTATTAGTCTAGTTGATACCATAGCAATAGCAGTAGCTGAAGGAACAGATAGGATTATAAACATAATACAAAGACTTTGTAGAGCTATCTRCAACGTACCTAGAAGAATAAGACAGGGCTTTGAAGCAGCTTTGCAATAA

>GX2016EU03

GCAGTGGGAATAGGAGCTATGATCTTTGGGTTCTTGGGAGCAGCAGGAAGCACTATGGGCGCGGCGTCAATAACGCTGACGGTACAGGCCAGGCAATTATTGTCTGGTATAGTGCAACAGCAAAGCAATTTGCTGAGGGCTATAGAGGCGCAGCAGCATATGTTGCAACTCACAGTCTGGGGCATTAAACAGCTCCAGGCGAGAGTCCTGGCTGTGGAAAGATACCTAAAAGATCAACGGCTCCTAGGACTTTGGGGCTGCTCTGGGAAAGTCATCTGCACCACTGCTGTGCCCTGGAACTCCACTTGGAGTAATAAATCTTTAGAACAGATTTGGAACAACATGACATGGGTAGAATGGGAGAGAGAAATTAACAATTATACAAGCCAAATATATGACATACTTACAGAATCGCAGAACCAGCAGGACAAGAATGAAAAGGATTTGTTGGAATTGGATAAATGGACAAGTCTGTGGAATTGGTTTAACATATCAAATTGGCTGTGGTATATAAAAATATTTATAATGATAGTAGGAGGTTTGATAGGTTTAAGAATAATTTTTGCTGTGCTTTCTATAGTAAATAGAGTTAGGCAGGGATACTCACCTTTGTCTCTCCAGATCCCTATCCGACAGCAGAGGGAACCCGACAGACTCGAAAGAATCGAAGAAGAAGGTGGCGAGCAAGGCAGAGACAGATCCGTGCGCTTAGTGAGCGGATTCTTAGCACTTGCCTGGGACGACCTACGGAGCCTGTGCCTCTTCAGCTACCACCTCTTGAGAGACTTCAGCTTGATTGTCGCGAGGACTGTGAA---------------------CAAGGGACTGAGACGGGGGTGGGAAGGCCTCAAATATCTGGGGAATCTTCTGGTATATTGGGGTCGGGAGCTGAAAATTAGTGCTATTTCTTTGCTTGATGCTATAGCAGTAACAGCAGCGGGGTGGACAGATAGGGTTATAGAGGTAGCACAAAGAGCTTGGAGAGCCTTTCTCCACATACCTAGAAGAATCAGACAAGGCTTAGAAAGGGCTTTGCAATAA

>GX2016EU04

GCAGTGGGAATAGGAGCTATGATCTTTGGGTTCTTAGGAGCAGCAGGAAGCACTATGGGCGCGGCGTCAATAACGCTGACGGTACAGGCCAGACAATTATTGTCTGGTATAGTGCAACAGCAAAGCAATTTGCTGAGGGCTATAGAGGCGCAGCAGCATCTGTTGCAACTCACAGTCTGGGGCATTAAACAGCTCCAGGCAAGAGTCCTGGCTGTGGAAAGATACCTAAAAGATCAAAAGCTCCTAGGACTTTGGGGCTGCTCTGGAAAAACCATCTGCCTCACTTCTGTGCCTTGGAACTCCACTTGGAGTAATAAATCTTATAAAGAGATTTGGGACAACATGACATGGATAGAATGGGAGAGAGAAATAAGTAATTACACAAACCAAATATATGATCTAATTACAGAATCACAGAACCAGCAGGAGAGGAATGAAAAAGATTTGTTAGAATTGGATAAATGGACAAGTCTGTGGAATTGGTTTGACATAACAAAATGGCTGTGGTATATAAAAATATTCATAATAATAGTAGGAGGTTTAATAGGTTTAAGAATAATTTTTGCTGTGCTTTCTATAGTAAATAGAGTTAGGCAGGGATACTCACCTTTGTCTCTCCAGATCCCTACCCATCAGCAGAGGGAAMCCGACAGACCCGAAAGAATCGAAGAAGAAGGTGGCGAGCAAGGCAGAGACAGATCCGTGCGATTAGTGAGCGGATTCTTAGCTCTTGCCTGGGACGATCTACGGAGCCTGTGCCTCTTCAGCTACCRCCTCTTGAGAAACTTCAGCTTGATTGCAGCGAGGACTGTGGAACT---------------------GGGACTGAGACGGGGGTGGGAAGGCCTCAAATACCTGKGGAATCTTCTGGTGTATTGGGGTCAGGAACTGAAAAATAGTGCTATTTCTTTGCTTGATGCYACAGCAATAGCAGTAGCAGGGGGGACAGATAGGGTTATAGAARTAGTACAAAGAGCTTGGAGAGCCATTATCCACATACCTAGAAGAATYAGACAGGGCGCAGAAAGGGCTTGGSTATAA

>GX2016EU05

GCAGTGGGACTAGGAGCTGTGTTCCTTGGGTTCTTGGGAGTAGCAGGAAGCACTATGGGCGCGGCGTCATTGATGCTGACGGTACAGGCCAGACAATTGCTGTCTGGTATAGTGCAACAGCAAAACAATTTGCTGAGAGCTATAGAGGCGCAACAGCATATGTTGCAACTCACGGTTTGGGGCATTAAACAGCTCCAAACAAGAGTCCTGGCTATAGAAAGATACCTACAGGACCAACGGCTCCTAGGGATTTGGGGCTGCTCTGGGAAAATCATCTGCACCACTGCTGTACCTTGGAACTCTAGTTGGAGTAACAAATCTCAAGCAGAGATTTGGGATAACATGACCTGGATGCAATGGGATAAAGAAATTGATAGGCACACAGACACAATATACAGGTTGCTTGAAGTCTCGCAAAACCAGCAGGAAAAGAATGAGAAAGACCTATTAGAATTGGACAAGTGGAACAATCTATGGAATTGGTTTGACATATCAAGTTGGCTGTGGTATATAAAAATATTCATAATGATAGTAGGAGGCTTGATAGGTTTAAGAATAATTTTTGCTGTGCTTTCTATAGTAAATAGAGTTAGGCAGGGATACTCACCTTTGTCGTTGCAGACCCTTATCCCGAACCCAGGGGGACCCGACAGGCTCGGAAGAATCGAAGAAGAAGGTGGAGAGCAAGGCAGAGACAGATCCATCAGATTAGTGAGCGGATTCTTAGCACTTGCGTGGGACGACCTACGGAACCTGTGCCTTTTCAGTTACCACCACTTGAGAGACTTTGCATTAGTGACAGCGAGAGTGGTGGAACTTCTGGGACGCAACAGCCTCAGGGGACTACAGAGGGGGTGGGAAGCCCTTAAATATCTGGGAAGTATTGTGCAGTACTGGGGTCAGGAGCTAAAAAAGAGTGCTATTAGTCTGCTTGATACCATAGCAATAGCAGTAGCTGAAGGAACAGATAGGATTATAGAATATATTCAAAGACTGTGTAGAGCTATCTGCAACATACCT-GAAGAAT-----AAGGTTTTGAAGCAGCTTTGCAATAA

>GX2016EU07

GCAGTGGGAATAGGAGCTATGATCTTTGGGTTCTTAGGAGCAGCAGGAAGCACTATGGGCGCAGCGTCAATAACGCTGACGGTACAGGCCAGACAATTATTGTCTGGTATAGTGCAACAGCAAAGCAATTTGCTGAGAGCTATAGAGGCGCAACAGCATATGTTGCAACTCACAGTCTGGGGCATTAAACAGCTCCAGGCAAGAGTCCTGGCTGTGGAAAGATACCTAAAGGATCAAAAGTTCCTAGGACTTTGGGGCTGCTCTGGAAAAATCGTCTGCACCACTAATGTGCCTTGGAACTCCACTTGGAGTAATAAAACATATAAACAGATTTGGGACAACATGACATGGATACAATGGGAGAGAGAAATTAGCAATTACACAGACATAATCTATGACCTACTTACAGAATCGCAGGACCAGCAGGATAAAAATGAAAAGGAATTGTTAGAGTTGAATAAATGGGCAAGTCTGTGGAGTTGGTTTGACATAACAAACTGGCTGTGGTATATAAAAATATTTATAATGATAGTAGGAGGTTTAATAGGTTTAAGAATAATTTTTGCTGTGCTTTCTATAGTAAATAGAGTTAGGCAGGGATACTCACCTTTGTCTTTCCAGATCCCCTCCCACCATCAGAGGGAACCAGACAGACTCGAAAGAATCGAAGAAGAAGGTGGCGAGCAAGGCAGAGACAGATCCGTGCGCTTAGTGACCGGATTCTTAGCACTTGCCTGGGACGACCTACGGAGCCTGTGCCTCTTCAGCTACCACCGCTTGAGAGACTTCATATTGCTTGTAACGAGGACTGTGGAACTTCTGGG------------CAAGGGACTGAGACGGGGGTGGGAAAGCCTCAAGTATCTGGGGAATCTTCTGTTATATTGGAGTCAGGAACTGAAAATTAGTGCTATTTCCTTGTTTAATACTACAGCAATAGCAGTAGCGGGGTGGACAGATAGGGTTATAGAAGTAACACAAAGAGCTTGGAGAGCTGTTCTCCACATACCTAGAAGAATCAGACAGGGTTTAGAAAGAGCTTTGCTATAA

>GX2016EU08

GCAGTGGGAATAGGAGCTGTGTTCCTTGGGTTCTTGGGAGTAGCAGGAAGCACTATGGGCGCGGCGTCAAYGGCGCTGACGGTACAGGCCAGACAACTGCTGTCTGGTATAGTGCAACAGCAAAGCAATTTGCTGAGGGCTATAGAGGCACAACAGCATATGTTGCAACTCACGGTCTGGGGCATTAAACAGCTCCAGACAAGAGTCCTGGCTATAGAAAGATACCTAAAGGATCAACAGCTCCTAGGGATTTGGGGCTGCTCTGGAAAACTCATCTGCACCACTGCTGTACCTTGGAACTCCAGTTGGAGTAACAAATCACAARCAAAAATTTGGAAKAACATGACCTGGATGCAATGGGATRAAGAAATTAGTAATTACACAGACACAATATACGGGTTGCTTGAAGTCTCGCAAAACCAGCAGGAAAGGAATGAGAAAGATCTATTAGCATTGGACAGTTGGAAAAATCTATGGAATTGGTTTGACATAACAAATTGGCTGTGGTATATAAAAATATTCATAATGATAGTAGGAGGCTTGATAGGTTTAAGAATAATTTTTACTGTGCTCTCTGTAGTAAATAGAGTTAGGCAGGGATACTCACCTTTGTCGTTGCAGACCCTTATCCCGAACCCAGGGGGACCCGACAGGCTCRGAAGAATCGAAGAAGAAGGTGGAGAGCAAGGCAGCGACAGATCCATTCGATTAGTCARCGGGTTCTTAGCACTTGCCTGGGACGATCTGCGGAACCTGTGCCTCTTCAGCTACCACCGCTTGAGAGACTGTGTATTAGTGACAACGAGAGTGGTGGAACTTCTGGGACGCAACAGCCTCAGGGGACTACAGAGGGGGTGGGAAGCCCTTAAATATCTGGGAAGTCTTGTGCAGTACTGGGGTCAGGAGCTAAAAAAGAGTGCTATTAGTCTGTTTGATACCATAGCAATAGTAGTAGCTGAAGGAACAGATAGGATTATAGAAGTAGGACAAAGACTTTGCAGAGCTATCTACAACATACCTAGAAGAATAAGACAGGGCTTTGAAGCAATTTTGCAATAA

>GX2016EU09

GCAGTGGGAATAGGAGCTATGATCTTTGGTTTCTTGGGAGCAGCAGGAAGCACTATGGGCGCAGCGTCAATAACGCTGACGGTACAGGCCAGACAATTATTGTCTGGTATAGTGCAACAGCAAAGCAATTTGCTGAGAGCTATAGAGGCGCAGCAGCATCTGTTGCAACTCACAGTCTGGGGCATTAAACAGCTCCAGGCAAGAGTCCTGGCTGTGGAAAGATACCTAAAAGATCAAAGGCTCCTAGGACTTTGGGGCTGCTCTGGAAAAATCATTTGCACCACTAATGTGTCCTGGAACTCCACTTGGAGTAATAAATCTTATGAAGAGATTTGGGGAAACATGACATGGATACAATGGGAGAAAGAAATTAACAATTACACAAATACAATCTATGACCTACTTACAGAATCGCAGAGCCAGCAGGAAAGAAATGAAAAGGATTTGTTAGAGTTGGACAAATGGACAAGTCTGTGGAATTGGTTTGACATAACAAACTGGCTGTGGTATATAAAAATATTCATAATGATAGTAGGAGGTTTAATAGGCTTAAGAATAATTTTTGCTGTGTTCTCTATAGTGAATAGAGTTAGGCAGGGATACTCACCTTTGTCGTTGCAGACCCTTATCCCGAACCCAGGGGGACCCGACAGGCTCGGAAGAATCGAAGAAGAAGGTGGAGAGCAAGACAGAGACAGATCCGTGCGATTAGTGACCGGATTCTTAGCACTTGCCTGGGACGACCTGCGGAACCTGTGCCTCTTCAGCTACCACCGCTTGAGAGACTTTGTATTAGTGACAACGAGAGTAGTGGAACTTCTGGGACGCAGCAGCCTCAAGGGACTGAGACGGGGGTGGGAAGGCCTCAAATATCTGGGGAATCTTCTGTTGTATTGGCTTCAGGAACTGAAAACTAGTGCTATCTCTTTGCTTGATGCTACAGCAATAGCAACAGCGGAGTGGACAGATAGGGTTATAGAAGTTGCACAAAGAGCTTGTAGAGCCATTCTTCACATACCTAGGAGAATCAGACAGGGCTTAGAAAGGACTTTGATATAA

>GX2016EU10

GCAGTAGGAATAGGAGCTATGATCTTTGGGTTCTTGGGAGCAGCAGGAAGCACTATGGGCGCAGCGTCAATAACGCTGACGGTACACGCCAGACAATTGTTGTCTGGTATAGTGCAGCAGCAAAGTAATTTGCTGAGAGCTATAGAGGCGCAACAGCATATGTTGCAACTCACAGTCTGGGGCATTAAACAGCTCCAGGCAAGAGTCCTGGCTGTGGAAAGATACCTAAAAGATCAAAGGTTCCTAGGACTTTGGGGCTGCTCTGGAAAAACCATCTGCACCACTAATGTGCCCTGGAACTCCACTTGGAGTAATAAATCTGTTGATGACATTTGGGGCAACATGACATGGGTAGAATGGGAGAGAGAAATTAGCAATTACACAAGCACAATCTATGATCTACTTACAGAATCACAGAACCAGCAGGACAAAAATGAAAAGGATCTGTTAGCATTGGACAAATGGGCAAGTCTGTGGAATTGGTTTGACATATCAAATTGGCTGTGGTATATAAGAATATTCATMATGATAGTAGGAGGCYTRATAGGTTTAAGAATARTTTTTRCTGTGCTYTCTATAGTRAATAGAGTTAGGCAGGGATACTCACCTCTGTCTTTTCAGACCCCTTCCCATCATCCGAGGGAACCCGGCAGGCCCGAAGGAATCGAAGAAGAAGGTGGAGAAGAAGGCAGAAACAGATCMGTGCGCTTAGTGAGCGGATTCTTTTCCCTTATCTGGGACGATCTACGGAGCCTGTGCCTCTTCAGCTACCACCGCTTGATAGACTTAGTCTCGATTGCAACGAGGACTGTGGAACTTCTGGGACGCAGCAGTCTCAAGGGACTGAGACGGGGGTGGGAAGGCCTCAAATATCTGGGGAATCTTCTGTTATATTGGGGACAGGAACTGAAAATTAGTGCTATTTCTTTGCTTGATGCTGCAGCAATAGCAACAGCGAGGTGGACAGATAGGGTTATAGAATTAGCACAAAGAGCTTGGAGAGCTGTTCTCCACATACCTAGAAGAATCAGACAAGGCTTAGAAAGGACTTTGGTATAA

>GX2016EU11

GCAGTAGGAATAGGAGCTATGATCTTTGGGTTCTTAGGAGCAGCAGGAAGCACTATGGGCGCAGCATCAATAACGCTGACGGTACAAGCCAGACAATTATTGTCTGGTATAGTGCAACAGCAAAGCAATTTGCTGAGAGCTATAGAGGCGCAGCAGCATATGTTGCAACTCACAGTCTGGGGCATTAAACAGCTCCAGGCAAGACTCCTGGCTGTGGAAAGATACCTAAAGGATCAAAAGTTCCTAGGACTTTGGGGCTGCTCTGGAAAAATCATCTGCACCACTAATGTGCCCTGGAACTCCACTTGGAGTAATAAATCTCTTGAACAAATCTGGGACAACATGACATGGGTAGACTGGGAGAGAGAAATTAGCAATTACACAGACATAATTTATGATCTACTTACAGAATCGCAAAACCAGCAGGACAAAAATGAAAAGGATTTGTTAGAATTGAACAGATGGGCAAGTCTATGGAGTTGGTTTGACATAACAAGA-GGCTGTGGTATATAAAAATATTTATAATGATAGTAGGAGGTTTAATAGGGTTAAGAATAATTTTTGCTGTACTTTCTATAGTAAATAGAGTTAGGGAGGGATACTCACCTTTGTCTTTCCAGACCCCCCTCCAGCATCAGAGGGAACCCGACAGGCCAGAAGGAATCGAAGAAGGAGGTGGCGAGCAAGGCAGAGACAGATCCGTGCGCTTAGTGAACGGATTCTTGGCAATTATCTGGGACGACCTACGGAGCCTGTGCATTTTCAGCTACCACCGCTTGAGAGACTTCATCTTGATTGCAACGAGGACTGTGGAACTTCTGGGACGCAGCAGTCTCAAGGGACTGAGACTGGGGTGGGAAGGCCTCAAATATCTGGGGAATCTTCTGTCATATTGGGGTCAGGAACTGAAAATTAGTGCTATTTCCTTGTTTGATGCTACAGCAATAGCAATAGCGGGGTGGACAGATAGGGTTATAGAAGTAGTACTAAGAGGTTGGAGAGCTCTTCTCCACATACCTAGAAGAATCAGACAGGGCTTCGAAAGGGCTTTGCTATAA

>GX2016EU12

GGAATAGGAATAGGAGCTGTGTTCCTTGGGTTCTTGGGAGTAGCAGGAAGCACTATGGGCGCGGCGTCAATGGCGCTGACGGGACAGGCCAGACAATTGCTGTCTGGTATAGTGCAACAGCAAAACAATTTGCTGAGGGCTATAGAGGCGCAACAGCATATGTTGCAACTCACGGTCTGGGGCATTAAACAGCTCCAAACAAGAGTCCTGGCTATAGAAAGATACCTACAGGATCAACAGCTCCTAGGGATTTGGGGCTGCTCTGGAAAAATCATCTGCACCACTGCTGTACCTTGGAACTCCAGTTGGAGTAACAAATCTCAAACAGAGATTTGGGGTAACATGACCTGGATGCAATGGGATAAAGAAATTAGTAATTACACAGCCACAATATACAGTTTGCTTGAAGACTCGCAAAACCAGCAGGAAAGGAATGAAAAAGATCTATTAGCATTGGACAATTGGAAAAATCTATGGAATTGGTTTGACATAACAAACTGGCTGTGGTATATAAAAATATTTATAATGATAGTAGGAGGCTTGATAGGTTTAAGAATAATTTTTGCTGTGCTCTCTATAGTGAATAGAGTTAGGCAGGGATACTCACCTTTGTCGTTGCAGACCCTTATCCCGAATCCAGGGGGTCCCGACAGGCTCGGAAGAATCGAAGAAGAAGGTGGAGAGCAAGACAGAGACAGATCCGTGAGATTAGTGACCGGATTCTTAGCACTTGCCTGGGACGACCTGCGGAACCTGTGCCTCTTCAGCTACCACCGCTTGAGAGACTTTGTATTAGTAACAACGAGAGTAGTGGAACTTCTGGGACGCAGCAGCCTCAGGGGACTACAGAGGGGGTGGGAAGCCCTTAAATATCTGGGAAGCCTTGTGCAGTACTGGGGTCAGGAGCTAAAAAAGAGTGCTGTTAGTCTGATTGATACCATAGCAATAGCAGTAGCTGAAGGAACAGATAGGATTATAGAAGTAGTACAAAGATTTTGTAGAGCTATATACAACATACCTACAAGAATAAGACAGGGCTTTGAAGCAAGTTTGCAATAA

>GX2016EU13

GCAGTGGGAATAGGAGCTATGATCTTTGGGTTCTTAGGAGCAGCAGGAAGCACTATGGGCGCGGCGTCAATAACGCTGACGGTACAGGCCAGACAATTATTGTCTGGTATAGTGCAACAGCAAAGCAATCTGCTAAGGGCTATAGAGGCGCAGCAGCATCTGTTGCAACTCACAGTCTGGGGCATTAAACAGCTCCAGGCAAGAGTCCTGGCTGTGGAAAGATACCTAAAAGATCAACAGCTCCTAGGACTTTGGGGCTGCTCTGGAAAAATCATATGCACCACTGCTGTGCCCTGGAACTTCACTTGGAGTAATAAATCTTATGATGAGATCTGGGACAACATGACATGGGTACAATGGGAGAGAGAAATTAGCAATTACACAAGCCAAATATATGGTATACTTACAGAATCGCAAAACCAGCAGGACCGGAATGAAAAGGATCTGTTAGAATTGGATAAATGGGCAAGTCTGTGGAATTGGTTTGACATAACAAATTGGCTGTGGTATATAAAAATATTTATAATAATCGTAGGAAGTTTAATAGGTTTAAGAATAATTTTTGCTGTGCTTTCCATAGTAAATAGAGTTAGGCAGGGGTACTCACCTTTGTCGTTCCAGATCCCTACCCATCACCAGAGGGATCCCGACAGACCAGAAGAAATAGAAGAAGGAGGTGGCGAGCAAGGCAGAGGCAGATCCGTGAGATTAGTGAGCGGATTCTTAGCACTTGCTTGGGACGATCTACGGAGCCTGTGCCTCTTCAGCTACCACCGCTTGAGAGACTTCATATTGATTGCATTGAGGACTGTGGAACTTCTGGGACACAGCAGTCTCAAGGGACTGAGACGGGGGTGGGAAGGTCTAAAATATCTGGGGAATCTTCTGGTATATTGGGGACAGGAACTAAAAATTAGTGCTATTTCTTTGCTTAATGCTACAGCAATAGCAGTAGCGGAGGGGACAGACAGGGTTATCGAAGTAGCACAGAGAGCTTGGAGAGCCTTTCTCCATATACCTAGAAGAATTAGACAGGGCTTAGAAAGGGCTTTGCAATAA

>GX2016EU14

GCAGTAGGAATAGGAGCTATGATCTTTGGGTTCTTAGGAGCAGCAGGAAGCACTATGGGCGCGGCGTCAATAACGCTGACGGTACAGGCCAGACAATTATTGTCTGGTATAGTGCAACAGCAAAGCAATTTGCTGAGAGCTATAGAGGCGCAGCAGCATCTGTTGCAACTCACAGTCTGGGGCATTAAACAGCTCCAGGCAAGAGTCCTGGCTGTGGAAAGATACCTAAGAGATCAAAAGTTCCTAGGACTTTGGGGCTGCTCTGGAAAAATCATCTGCACCACTGCTGTGCCCTGGAACTCCACTTGGAGTAATAAATCTTATAACGAGATTTGGGACAACATGACATGGATACAATGGGAGAGAGAAATCAGTAATTTTACAGACCAAATATATCAGCTACTTACAGACTCGCAGAACCAGCAGGATAARAATGAAAAGGATTTGTTAGAATTGGATAAGTGGACAAGCCTGTGGAATTGGTTTGACATAACAAGATGGCTGTGGTATATAAAAATATTTATAATGATAGTAGGAGGTTTAATAGGTTTAAGAATCATTTTTGCTGTGCTTTCTATAGTAAATAGAGTTAGGCAGGGATACTCACCTTTGTCTCTCCAGATCCCTCCCCTACAGCAGAGGGAACCCGACAGGCCCGAAGGAATCGAAGAAGAAGGTGGCGAGCAAGGCAAAGACAGATCCGTGAGATTAGTGACCGGATTCTTCGCTCTTGCCTGGGACGATCTCCGGAGCCTGTGTCTTTTCAGCTACCGCCTCTTGAAAGACTTCAGCTTGATTGTAGCGAGGACTGTGAA---------------------CAAGGGACTGAGACGGGGGTGGGAAGGCCTCAAATATCTGGGGAATCTGCTGATATATTGGAGTCAGGAACTAAAGACTAGTGCTATTTCTTTGCTTGATACTATAGCAATAGCAGTAGCAGGGTGGACAGATAGGGTTATAGAAGTAGCACAAATAGCTTGGAGGGCCATTCTCCACATACCTAGAAGAATCAGACAGGGCTTAGAAAGGTCTTTGCTATAA

>GX2016EU15

GCAGTGGGAATAGGAGCTATGATCTTTGGGTTCTTGGGAGCAGCAGGAAGCACTATGGGCGCAGCGTCAATAACGCTGACGGTACAGGCCAGACAATTATTGTCTGGTATAGTGCAACAGCAAAGCAATTTGCTGAGAGCTATTGAGGCGCAGCAGCATATGTTGCAACTCACAGTCTGGGGCATTAAACAGCTCCAGGCAAGAGTCCTGGCTGTGGAAAGATACCTAAAAGATCAAAAGTTCCTAGGACTTTGGGGCTGCTCTGGAAAAATCATCTGCACCACTGCTGTGCCCTGGAACTCCACTTGGAGTAATAAGTCTTATGAAGAAATTTGGGACAACATGACATGGATAGAATGGGAAAAAGAAATTAGCAATTATACTAACATAATATATGACATACTTACAAAATCGCAGGACCAGCAGGACAGGAATGAAAAGGATTTGTTAGAATTGGATAAATGGACAAGTCTGTGGAGTTGGTTCAGCATAACAAATTGGCTGTGGTATATAAAAATATTTATAATAATAGTAAGAGCTTTAATAGGTTTAAGAATAGTTTTTGCTGTGCTTTCTATAGTAAATAGAGTTAGGCAGGGATACTCACCTCTGTCTCTCCAGATCCCTACCCATCAGCAGAGGGAACCAGACAGACCCGAAAGAATCGAAGAAGAAGGTGGCGAGCAAGGCAGAGACAGATCCGTGCGCTTAGTGACCGGATTCTTAGCTCTTGCCTGGGACGATCTGCGGAGCCTGTGCCTCTTCAGCTACCACCTCTTGAGAGACTTCAGCTTGATTGCAGCGAGGACTGTGGA---------------------CCAGGGACTGAGACGGGGGTGGAAAGTCCTCAAATATCTGGGGAATCTTCTGTCATATTGGGGTCAGGAACTAAAGAGTAGTGCTATTTCTTTGCTTGATACTACAGCAATAGCAGTAGCGGGGTGGACAGATAGGATTATAGAAGTAGCACAAGGAGCTTGGAGAGCCATTCTCCACATACCTAGAAGAAT---------------------------ATAA

>GX2016EU17

GCAGTCGGAATAGGGGCTATGATCTTTGGGTTCTTAGGAGCAGCAGGAAGCACTATGGGCGCGGCGTCAATAACGCTGACGGTACAGGCTAGACAATTATTGTCTGGTATAGTGCAACAGCAAAACAATTTGCTGAGGGCTATAGAGGCGCAACAGCACCTGTTGCAACTCACAGTCTGGGGCATTAAACAGCTCCAGGCAAGAGTCCTGGCTGTGGAAAGATACCTAAAAGATCAAAAGTTCCTAGGACTTTGGGGCTGCTCTGGAAAAACCATCTGCCCCACTGCTGTGCCCTGGAACTCCTCCTGGAGTAATAAATCTCTTGAAGAGATTTGGAACAACATGACATGGATACAATGGGAGAGAGAAATTAGCAATTATACAGACCTAATATATGAAATACTTACAGACTCGCAGAACCAGCAGGACAGGAATGAAAAGGATCTATTAGAATTGGATAAATGGACAAGTCTGTGGAATTGGTTTAACATAACAAATTGGCTGTGGTATATAAAAATATTTATAATGATAGTAGGAGGTTTAATAGGTTTAAGAATAATTTTTGCTGTGCTTTCTGTAGTAAATAGAGTTAGGCAGGGATACTCACCTTTGTCTTTCCAGATCCCTATCCATCAGCAGAGGGAACCCGACAGACCCGAAAGAATCGAAGAAGAAGGTGGCGAGCAAGGCAGAGACAGATCCGTGCGATTAGTGAGCGGGTTCTTAGCGCTTGCGTGGGACGATCTACGGAGCCTGTGCCTCTTCAGCTACCGCCTCTTGAGAAACTTCAGCTTAATTGCAGCGAGGACTGTGGAACTTCTGGGACACAGCAGTCTCAAGGGACTGAGACGGGGGTGGGAAGGCCTCAAATATCTGGGGAATCTTCTGTTGTATTGGGGTCAGGAACTAAAAATTAGTGCTATTTCTTTGCTTGATGCTACAGCAGTAGCAGTAGCGGGGTGGACAGATAGAGTTATAGAAGTAGCACAAAGAGCTTGGAGAGCCATTCTGCACATACCTAGAAGAATCAGACAAGGCTTAGAAAGGTCTTTGCTATAA

>GX2016EU18

GCAGTGGGAATAGGAGCTTTGTTCCTTGGGTTCTTGGGAGCAGCAGGAAGCACTATGGGCGCAGCGTCAATGACGCTGACGGTACAGGCCAGACAATTATTGTCTGATATAGTGCAGCAGCAGAACAATTTGCTGAGGGCTATTGAGGCGCAACAGCATCTGTTGCAACTCACAGTCTGGGGCATCAAACAGCTCCAGGCAAGAATCCTGGCTGTGGAAAGATACCTAAAGGATCAACAGCTCCTGGGGATTTGGGGTTGCTCTGGAAAACTCATTTGCACCACTGCTGTGCCTTGGAATGCTAGTTGGAGTAATAAATCTCTGGAACAGATTTGGAATAACATGACCTGGATGGAGTGGGACAGAGAAATTAACAATTACACAAGCTTAATACACTCCTTAATTGAAGAATCGCAAAACCAGCAAGAAAAGAATGAACAAGAATTATTGGAATTAGATAAATGGGCAAGTTTGTGGAATTGGTTTAACATAACAAATTGGCTGTGGTATATAAAATTATTCATAATGATAGTAGGAGGCTTGGTAGGTTTAAGAATAGTTTTTGCTGTACTTTCTATAGTGAATAGAGTTAGGCAGGGATATTCACCATTATCGTTTCAGACCCACCTCCCAATCCCGAGGGGACCCGACAGGCCCGAAGGAATAGAAGAAGAAGGTGGAGAGAGAGACAGAGACAGATCCATTCGATTAGTGAACGGATCCTTAGCACTTATCTGGGACGATCTGCGGAGCCTGTGCCTCTTCAGCTACCACCGCTTGAGAGACTTACTCTTGATTGTAACGAGGATTGTGGAACTTCTGGGACGCA---------------------GGGGGTGGGAAGCCCTCAAATATTGGTGGAATCTCCTACAGTATTGGAGTCAGGAACTAAAGAATAGTGCTGTTAACTTGCTCAATGCCACAGCCATAGCAGTAGCTGAGGGGACAGATAGGGTTATAGAAGTATTACAAGCAGCTTATAGAGCTATTCGCCACATACCTAGAAGAATAAGACAGGGCTTGGAAAGGATTTTGCTATAA

>GX2016EU22

GCAGTAGGACTAGGAGCTGTGTTCCTTGGGTTCTTGGGAGCAGCAGGAAGCACTATGGGCGCGGCGTCAATAACGCTGACGGTACAGGCCAGACAATTGTTGTCTGGTATAGTGCAACAGCAAAGCAATTTGCTGAGAGCTATAGAGGCGCAACAGCATATGTTGCAACTCACGGTCTGGGGCATTAAGCAGCTGCAGGCAAGAGTCCTGGCTATAGAAAGATACCTACAGGATCAACAGCTCCTAGGGCTTTGGGGCTGCTCTGGAAAACTCATCTGCACTACTGCTGTACCTTGGAACTCCAGTTGGAGTAACAAATCTCAACAAGAGATTTGGGATAACATGACCTGGATGCAGTGGGAAAGGGAAATTAGTAATTACACAGAAACAATATACAACTTGCTTGAAGTCTCGCAAAACCAGCAGGAAACAAATGAAAAGGATTTATTAGCATTGGACAGTTGGAATAATCTATGGAGTTGGTTTAACATATCAAATTGGCTGTGGTATATAAAAATATTCATAATGATAGTAGGAGGCTTGATAGGTTTAAGAATAATTTTTGCTGTGCTTTCTATAGTAAATAGAGTTAGGCAGGGATACTCACCTTTGTCGTTCCAGATCCTTACCCCGAACCCAGAGGGACCAGGCAGGCTCGGAAGAACCGAAGAAGAAGGTGGAGAGCAAGACAAAGGCAGATCCATCAGATTAGTGAACGGATTCTTAGCACTTGCCTGGGACGACCTGCGGAACCTGTGCCTCTTCAGTTACCACCGCTTGAGAGACTTAATATCAGTGACAGCGAGAGGGGTGGAACTTCTGGGACGCAACAGCCTCAAGGGACTACGGAGGGGGTGGGAAGCCCTTAAATATCTGGGAAGTCTTGGGCAGTATTGGGCTCTAGAGCTAAAAAAGAGTGCTATTAGTCTGTTTGATGTCATAGCAATAGCAGTAGCTGAAGGAACAGATAGGATTATAGCCATAGTACAAAGAATTTGTAGAGCTATCCGCAACGTACCTAGAAGAGTAAGACAGGGCTTAGAAGCAACTTTGCAATAA

>GX2016EU23

GCAGCA---ATAGGAGCTGTGTTCCTTGGGTTCTTGGGAGCTGCAGGAAGCACTATGGGCGCGGCGTCAATGACGCTGACGGTACAGGCCAGACAATTGCTGTCTGGTATAGTGCAACAGCAAAGCAATTTGCTGAAGGCTATAGAGGCGCAACAGCATATGTTGCAACTCACGGTCTGGGGCATTAAGCAGCTCCAGACAAGAGTCCTGGCTATAGAAAGATACCTAAAGGATCAACAGCTCCTAGGAATTTGGGGCTGCTCTGGAAAACTCATCTGCACCACTACTGTACCTTGGAACTCCAGTTGGAGCAACAAATCTCAAACAGAGATTTGGAATAACATGACATGGATGCAGTGGGATAAAGAAATTAATAATTACACAAACACAATATACAGTTTGCTTGAAGAATCACAAAACCAGCAGGAAAAGAATGAGAAAGATCTATTAGCATTGGACAGTTGGAAAAATCTATGGAATTGGTTTAGCATAACAAATTGGCTGTGGTACATAAAAATATTCATAATGATAGTAGGAGGTTTGATAGGTTTAAGAATAATTTTTGCTGTGTTCTCTATAGTGAATAGAGTTAGGCAGGGATACTCACCTTTATCGTTGCAGACCCTTATCCCGAACCCAGTGGAACCCGACAGACCCGAAAGAATCGAAGAAGGAGGTGGAGAGCAAGGCAGAGACAGATCCGTGAGATTAGTGAGCGGGTTCTTGCCACTTGCTTGGGACGATCTACGGAGCCTGTGCCTCTTCCTCTACCACCGCTTGAGCGACTTCACATTGATTGCAGCGAGGACTGTGGAACTTCTGGGAC------------AGGGACTGAGACGGGGGTGGGAAGGCCTCAAATATCTGGGGAATCTTCTGTTATATTGGAGTCAGGAACTGAAAAATAGTACTATTTCTTTGCTTAATACTATAGCAATAGCAGTAGCAGGGTGGACAGATAGCCTTATAGAAGCAGCACAAGGAGCTGGGAGAGCTATTCTCCACATACCTAGAAGAATCAGACAGGGCTTAGAAAGGGCTTTGCTATAA

>BJ2015EU01

GCAGTGGGACTAGGAGCTATGATCTTTGGGTTCTTAGGAGCAGCAGGAAGCACTATGGGCGCAGCGTCAATAACGCTGACGGTACAGGCCAGACAATTATTGTCTGGTATAGTGCAACAGCAAAGCAATTTGCTGAGAGCTATAGAGGCGCAGCAGCATCTGTTGCAACTCACAGTCTGGGGCATTAAACAGCTCCAGGCAAGAGTCCTGGCTGTGGAAAGATACCTAAAAGATCAAAAGTTCCTAGGACTTTGGGGCTGCTCTGGAAAAATCATCTGCACCACTAATGTGCCCTGGAACTCCACTTGGAGTAATAAATCTTATAAAGAGATTTGGGACAACATGACATGGATAGAATGGGAGAGAGAAATTAGCAATTACACAGGCATAATCTATGACCTACTTACAGAATCGCAGAACCAGCAGGAAAGAAATGAAAAGGATTTGTTAGAATTGGACAAATGGGCAAGTCTGTGGAATTGGTTTGACATAACACAATGGCTGTGGTATATAAGAATATTTATAATGATAGTAGGAGGGTTAATAGGTTTAAGAATCATTTTTGCTGTGCTTTCTATAATAAATAGAGTTAGGCAGGGATACTCACCTTTATCTCTCCAGACCCCTCTCCACCAGCAGAGGGAACCCGACAGGCCCGAAGGAATCGAAGAAGAAGGTGGCGAGCAAGGCAGAGACAGATCCGTGCGATTAGTGAGCGGATTCTTAGCTCTTGTGTGGGACGATCTACGGAACCTGTGCCGCTTCAGCTACCACCGCTTGAGAGACTTCATCTTGATTGCAACGAGGACTGTGGAACTTCTGGG------------CAAGGGACTGAGACGGGGGTGGGAGGGCCTCAAATATCTGGGGAATCTCCTGTTATATTGGATTCAGGAACTAAAAACTAGTGCTATCTCTTTGCTTGATGCTACAGCAATAGCAACAGCGGGGTGGACAGATAGGGTTATAGAAATAGCACAAAGAGCTTGGAAAGCTTTTCTCCACATACCTAGAAGAATCAGACAGGGCTTAGAAAGGGCTTTGCAATAA

>BJ2015EU02

---------------------------------------------------------------------------------------------------------------------------------------------------------------CTATTGTA-------------------------CTCCAG-------------------------------------------------------------------------------------------------------CTGGTT-----------------------------------------------------------------------------------ATGCAATTCTTA---GAAGAATCGCAAATCCAGCAGGAACAGAATGAAAAAGATTTATTGGCATTGGACAGTTGGAACAATCTATGGAATTGGTTTGACATATCAAATTGGCTGTGGTATATAAAAATATTCATAATAGTAGTAGGGGGCTTGATAGGTTTAAGAATAGCTTTTGCTGTACTCTCTATAGTAAATAGAGTTAGGCAGGGATACTCACCATTGTCATTTCAGACCCATACCCGGAACCCAGGGGCACCCGACAGGCTCGGAAGAATCGAAGAAGGAGGTGGAGAGCAAGACAACGGCGGATCCATTCGATTAGTGAACGGATTCTTAGCTCTTGCCTGGGACGACCTGAGGAGCCTGTTCCTCTTCAGCTACCACCAATTGAGAGACTTCATATTGGTGACAGCGAGAACGGTGGAGCTTCTGGGACGCAACAGCCTCAAGGGACTACAGAGGGGGTGGGAAGCCCTTAAATATCTGGGAAGTCTTGTGCAGTACTGGGGTCAGGAGCTGAAAAAGAGTGCTATTAGTTTGTTTGATACCATAGCAATAGCAGTAGCTGAAGGAACAGATAGGATTGTAGAATTAGTACAAAGACTTTGTAGAGCTATCTGCAACGGTCCTAGAAGAATAAGACAGGGCTTGGAAGCAGCTTTGCAATAA

>BJ2015EU03

GCAGTGGGACTAGGAGCTATGATCTTTGGGTTCTTAGGAGCAGCAGGAAGCACTATGGGCGCGGCGTCAATAACGCTGACGGTACAGGCCAGACAATTATTGTCTGGTATAGTGCAACAGCAAAGCAATTTGCTGAGGGCTATTGAGGCGCAGCAGCATCTGTTGCAACTCACAGTCTGGGGCATTAAACAGCTCCAGGCAAGAGTCCTGGCTGTGGAAAGATACCTAAAGGATCAAAAGTTCCTAGGACTTTGGGGTTGCTCTGGAAAAATCATCTGCACCACTAATGTGCCTTGGAACTCCACTTGGAGTAATAAAACTCTTGATGAGATTTGGAACAACATGACATGGATAGAATGGGAGAGAGAAATTAGCAATTACACAAACCAAATCTATCAGCTACTTACGGAATCGCAGAACCAGCAGGACAAAAATGAAAAGGATTTGTTAGAATTGGACAAATGGGCAAGTCTGTGGAATTGGTTTGACATAACAAGATGGCTGTGGTATATAAAAATCTTTATAATGATAGTAGGGGGTTTAATAGGTTTAAGAATAATTTTTGCTGTACTTTCTATAGTAAATAGAGTTAGGCAGGGATACTCACCTTTGTCTTTACAGACCCTTACCCATCACCAGAGGGAACCCGACAGGCCCGAAGGAATCGAAGAAGGAGGTGGCGAGCAAGGCAGAGACAGATCCGTGCGATTAGTGAGCGGATTCTTAGCTCTTGCGTGGGACGACCTACGGAGCCTGWGCCTCTTCCTTTACCACCACTTGAGAGACTTCATCTGGATTGCAACGAGGACTGTGGG---------------------CAAGGGACTGAGACGGGGGTGGGAAGGCCTCAAATATCTGGGGAATCTTCTGGCATATTGGAGTCAGGAACTGAAGAKTAGTGCTATTTCTTTGCTTGATACTATAGCAATAGCAACAGCGGGGTGGACAGATAGGGTTATAGAAGCAGCACAAASAGCTTGGAGAGCTTTTATCCACATACCTAGAAGAATCAGACAGGGCTTGGAAAGGGCTTTGCTATAA

>BJ2015EU04

GCAGTGGGAATAGGAGCTGTGTTCCTTGGGTTCTTGGGAGTAGCAGGAAGCACTATGGGCGCGGCGTCAATGACGCTGACGGTACAGGCCAGACAATTGCTGTCTGGTATAGTGCAACAGCAAAGCAATTTGCTGAGGGCTATAGAGGCGCAACAGCATATGTTGCAACTCACAGTCTGGGGCATTAAACAGCTCCAAACAAGAGTCCTGGCTATAGAAAGATACCTAAGGGATCAACAGCTCCTAGGGATTTGGGGCTGCTCTGGAAAACTCATCTGCACCACTGCTGTACCTTGGAATTCCAGTTGGAGTAACAAATCYCAACCAGAAATTTGGGAGAACATGACCTGGATGCAATGGGATAAAGAAATTAGTAATTACACAAACACAATATATAGGTTGCTTGAAGACTCGCAAAACCAGCAGGAAAGGAATGAAAAAGATCTATTAGCATTGGACAGTTGGAACAATCTATGGAGTTGGTTTGACATATCAAATTGGCTGTGGTATATAAGAATATTCATAATGATAGTAGGAGGCTTGATAGGTTTAAGAATAATTTTTGCTGTGCTCTCTATAGTGAATAGAGTTAGGCAGGGATACTCACCTTTGTCGTTGCAGACCCTTATCCCGAACCCAGGGGGACCAGACAGGCTCGGAAGAATCGAAGAAGAAGGTGGAGAGCAAGACAGAGACAGATCCATTCGATTAGTGAACGGATTCTTAGCACTTTTCTGGGACGATCTGCGGAACCTGTGCCTCTTCAGCTACCACCGCTTGAGAGACTTTGTATTAGTGACAGCGAGAGTGGTGCAACT---------------------GGGACTACAGAGGGGGTGGGAAGTCCTTAAATATCTGGGAAGTCTTGTGCAGTACTGGGGACAGGAGCTAAAAAAGAGTGCTATTAGTCTGCTTGATATCATAGCAATAGCAGTAGCTGAAGGAACAGATAGGATTATAGAAGTAGGACAAAGACTTTGTAGAGCCATCTACAACATACCTACAAGAATAAGACAGGGCTTTGAAACAGCTTTGCAATAA

>BJ2015EU06

GCAGTGGGAATAGGAGCTATGATCTTTGGGTTCTTAGGAGCAGCAGGAAGCACTATGGGCGCAGCGTCAATAACGCTGACGGTACAGGCYAGACAATTATTGTCTGGTATAGTGCAACAGCAAAACAATTTGCTGAGAGCTATAGAGGCGCAGCAGCATCTGTTGCAACTCACAGTCTGGGGCATTAAACAGCTCCAGGCAAGAATCCTGGCTGTGGAAAGATACCTAAAAGATCAACAGTTCCTAGGACTTTGGGGCTGCTCTGGAAAAACCATCTGCACCACTAATGTGCCCTGGAACTCCACTTGGAGCAATAAATCTTATGATGAGATTTGGCAAAACATGACATGGATAGAATGGGAGAGGGAAATTRGCAAATACACAAACAACATTTTTGAGCTACTTAYAGAATCGCAGAACCAGCAGGACAGAAATGAAAARGATTTATTAGAATTGGACAAATGGGCAAGTCTGTGGAATTGGTTTGACATAACAAACTGGCTGTGGTATATAAAAATATTCATAATGATAGTAGGAGGTTTAATAGGTTTAAGAATAATTTGTGCTGTGTTTTCTATAGTGAATAGAGTTAGGCAGGGWTACTCACCTTTGTCGTTCCAGACCCTTCCCCGCCACCAGAGGGAACCCGACAGGCCCGAAGGAATCGAAGAAGGAGGTGGCGAGCAAGGCAGAGACAGATCCGTACGCTTAGTGAGCGGGTTCTTAGCAATTTTCTGGGACGACCTCCGGAGCCTGTGCCTCTTCAGCTACCACCGCTTGAGAGACTTCAGCTTGATTGCAGCGAGGACTGTGGAACTTCTGGGACGCAGCATTCTCCAGGGACTGAGACGGGGGTGGGAAGGCCTCAAATATCTGGGGAATCTCCTGTTATATTGGGGTCAGGAACTGAAAATTAGTGCTACTTCTTTGTTTGATGCTACAGCAATAGCAATAGCAGGGTGGACAGATAGGGTTATAGAAGCAGTACAAAGAGCTGGCAGAGCTATCCTCCACATACCTAGGAGAGTCAGACAGGGCTTAGAAAGGGCTTTGCTATAA

>BJ2015EU08

GCAGTGGGAATAGGAGCTGTGTTCCTTGGGTTCTTGGGAGTAGCTGGRAGCACTATGGGCGCGGCGTCAATGACGCTGACGGTACAGGCCAGACAATTGCTGTCTGGTATAGTGCAACAGCAAAGTAATTTGCTGAGAGCTATAGAGGCGCAACAGCATATGTTGCAACTCACAGTCTGGGGCATTAAGCAGCTCCAGGCAAGAGTCCTGGCTATAGAAAGATACCTAAAGGATCAACAGCTCCTAGGGATTTGGGGCTGCTCTGGAAAACTCATCTGCACTACTGCTGTACCTTGGAATTCCACTTGGAGTAACAAAACTTACACAGAGATTTGGGATAACATGACCTGGATGCAATGGGATAAGGAGATTGATAATTACACAAACACAATATACCAATTGCTTGAAGAATCGCAAAARCAGCAGGAAAGAAATGAACAAGATCTATTAGCATTGGACAGTTGGAAAAATCTATGGAACTGGTTTAACATAACAAATTGGCTGTGGTATATAAAAATATTCATAATGATAGTAGGAGGCATGRTAGGTTTAAGAATAATTTTTGCTGTGCTTTCTGTAGTGAATAGAGTTAGGCAGGGCTACTCACCTTTGTCGTTTCAGACCCTTACCCCGAACCCCAGGGGACTCGACAGGCTCGGAAGAATCGAAGAAGAAGGTGGAGAGCAAGACAAAGACAGATCCGTTCGATTAGTCAGCGGATTCTTGACGCTTGCCTGGGACGATCTGCGGAACCTGTGCCTCTTCAGCTACCACCGATTGAGAGACTTCATATCAGTGGCAGCGAGAGTGGTGGAACTTCTGGGACGCACCAGTCTCAAGGGACTACAGAGGGGGTGGGAAGCCCTCCAATATCTGGGGAGTCTTGTGCAGTACTGGGGTCAGGAGCTAAAGAAGAGGGCTATTAGTCTGATTGATACCATAGCAATAGCAGTAGCTGAAGGAACAGATAGGATTATAGAACTAGTACAAGGATTTTTTAGAGCCATCTACAACATACCTAGAAGAATAAGACAGGGCTTTGAAGCAGCTTTGCAATAA

>BJ2015EU09

GCAGTGGGAATAGGAGCTATGATCTTTGGGTTCTTAGGAGCAGCAGGAAGCACTATGGGCGCAGCGTCAATAGCGCTGACGGGACAGGCCAGACAATTATTGTCTGGTATAGTGCAACAGCAAAGCAATTTGCTGAGGGCTATAGAGGCGCAGCAGCATCTGTTGCAACTCACAGTCTGGGGCATTAAACAGCTCCAGGCAAGAGTCCTGGCTGTGGAAAGATACCTAAAAGATCAACAGTTCCTAGGACTTTGGGGCTGCTCTGGAAAAATCATCTGCACCACTAATGTGCCTTGGAACTCCACTTGGAGTAATAAATCTCATGACGAGATTTGGAACAACATGACATGGATAAAATGGGAGAAAGAAATAAGCAATTACACAAACACAATCTATGAGCTACTTACAGAATCGCAGGACCAGCAGGAAAGAAATGAAAAGGATTTGTTAGAGTTAGATAAATGGACAAGTCTGTGGAATTGGTTTGACATATCAAACTGGCTGTGGTATATAAAAATATTTATAATGATAGTAGGAGGTTTAATAGGTTTAAGAATAATTTTTGCTGTGCTTTCTATAGTAAATAGAGTTAGGCAGGGATACTCACCTTTGTCTTTCCAGACCCCTACCCGTCATCAGAGGGAACCCGRCAGGCCCGAARGAATCGAAGAAGAAGGTGGCGAGCAAGGCAGAGGCAGATCAGTGCGCTTAGTGAGCGGATTCTTAGCACTTGCCTGGGACGATCTACGGAGCCTGTGCCTCTTCAGCTACCACCGCTTGAGAGACTTAATCTGGATTGCAACGAGGACTGTGGAACTTCTGGG------------CAAGGGACTGAGACGGGGGTGGGAACACCTCAAATATCTGGGGAATCTTCTGTTATATTGGGGTCAGGAACTGAAAATTAGTGCTATTTCTTTGTTTGATGCTACAGCAATAGCAATAGCAGGGTGGACAGATAGGGTTATAGAAGTAGCACAAACAGCCTGGAGGGCTATTCTCCACATACCTAGAAGAATCAGACAGGGCTTAGAAAGGGCTTTGCTATAA

>BJ2015EU11

GCAGTGGGAATAGGAGTTATGATCTTTGGGTTCTTAGGAGCAGCAGGAAGCACTATGGGCGCAGCGTCACTAACGCTGACGGTACGGGCCAGACAATTATTGTCTGGTATAGTGCAACAGCAAAGCAATTTGCTGAGAGCTATAGAGGCGCAGCAGCATCTGTTGCAACTCACAGTCTGGGGCATTAAACAGCTCCAGGCAAGAGTCCTGGCTGTGGAAAGATACCTAAAAGATCAAAAGCTCCTAGACCTTTGGGGCTGCTCTGGAAAAACCATCTGCACCACTAATGTGCCCTGGAACTCCACTTGGAGTAATAAATCTTATGAAGAGATTTGGAACAACATGACATGGATAGAATGGGAGAGAGAAATTAGCAATTACACAGACAAAATATATGAACTACTTATGGAATCACAGAACAAGCAGGAGAGCAATGAAAAGGAATTGTTAGAATTGGACAAATGGRCAAGTCTGTGGAATTGGTTTGACATAACAAACTGGCTGTGGTATATAAAAATATTTATAATGATAGTAGGAGGGTTAATAGGTTTAAGAATAATTTTTGCTGTGCTTTCTATAGTAAATAGAGTTAGGCAGGGATACTCACCTTTATCTTTCCAGATCCCTTTCCATCATCAGAGGGAACCCGACAGRCCCGAAGGAATCGAAGAAGAAGGTGGCGAGCGAGGCAGAGACAGATCCGTGAGATTAGTCAGCGGATTCTTGACTCTTGCGTGGGAAGATCTGCGGAGCCTGTGCCTCTTCAGCTACCGCCGCTTGAGAGACTTAATCTTGATTGCAACGAGGACTGTGGAACTTCTGGG------------CAAGGGACTGAGACGGGGGTGGGAAGGCCTCAAATATCTGGGGAATCTTCTGTTATATTGGGGTCAGGAACTGAAAACTAGTGCTATCTCTTTGCTTGATGCTATAGCAATAACAATAGCGGGGTGGACAGATAGGGTAATAGAAGTTGCACAAAGAGCTTGGAGAGCTTTGCTCCACATACCGAGAAGAATCAGACAGGGCTTCGAAAGGGCTTTGCTATAA

>BJ2015EU12

GCAGTGGGAATAGGAGTTATGATCTTTGGGTTCTTAGGAGCAGCAGGAAGCACTATGGGCGCAGCGTCACTAACGCTGACGGTACAGGCCAGACAATTATTGTCTGGTATAGTGCAACAGCAAAGCAATTTGCTGAGAGCTATAGAGGCGCAGCAGCATCTGTTGCAACTCACAGTCTGGGGCATTAAACAGCTCCAGGCAAGAGTCCTGGCTGTGGAAAGATACCTAAAAGATCAAAAGCTCCTAGACCTTTGGGGCTGCTCTGGAAAAATCATCTGCACCACTACTGTGCCCTGGAACTCCACTTGGAGTAATAAATCTTATGAAGAGATTTGGAACAACATGACATGGATAGAATGGGAGAGAGAAATTAGCAATTACACAAACAAAATCTATGATCTACTTATGGAATCGCAGAACCAGCAGGAGACAAATGAAAAGGATTTGTTAGAATTGGACAAATGGGCAAGTCTGTGGAATTGGTTTGACATAACAAACTGGCTGTGGTATATAAAAATATTTATAATGATAGTAGGAGGGTTAATAGGTTTAAGAATAATTTTTGCTGTGCTTTCTATAGTAAATAGAGTTAGGCAGGGATACTCACCTTTATCTTTCCAGACCCCTTTCCATCATCAGAGGGAACCCGACAGGCCCGAAGGAATCGAAGAAGAAGGTGGCGAGCGAGGCAGAGACAGATCCGTGCGATTAGTGAGCGGGTTCTTAGCTCTTGCGTGGGACGATCTGCGGAGCCTGTGCCTCTTCAGCTACCRCCGCTTGAGAGACTTAATCTTGATTGCAACGAGGACTGTGGAACTTCTGGG------------CAAGGGACTGAGACGGGGGTGGGAGGGCCTCAAATATCTGGGGAATCTTCTGTTRTATTGGGGTCAGGAACTGAAAACTAGTGCTATCTCTTTGCTTGATGCTATAGCAATAACAACAGCGGGGTGGACAGATAGGGTAATAGAAGTTGCACAAAGAGCTTGGAGAGCTTTTCTCCACATACCGAGAAGAATCAGACAGGGCTTCGAAAGGGCTTTGCTATAA

>BJ2015EU13

GCAGTGGGAATAGGAGCTGTGTTCCTTGGGTTCTTGGGAGTAGCAGGAAGCACTATGGGCGCGGCGTCAGTGGCGCTGACGGTACAGGCTAGACAATTGCTGTCTGGTATAGTGCAACAGCAAAGCAATTTGCTGAGGGCTATAGAGGCGCAACAGCATATGTTGCAACTCACGGTCTGGGGCATTAAACAGCTCCAAACAAGAGTCCTGGCTATAGAAAGATACCTAAAGGATCAACAGCTCCTAGGGATTTGGGGCTGCTCTGGAAAACTCATATGCACCACTGCTGTACCTTGGAACTCCAGTTGGAGTAACAAAACTCAAAGMGAGATTTGGGATAACATGACCTGGATGCAATGGGATAAAGAAATTAGTAATTACACAGACATAATATACAAGTTGCTTGAAGTCTCGCAAAACCAGCAGGAAAGAAATGAGAAAGATTTATTGGCATTGGACAGTTGGAAAAATCTATGGAATTGGTTTGACATAACAAATTGGCTGTGGTATATAARAATATTCATAATGATAGTGGGAGGCTTGATAGGTTTAAGAATAATTTTTGCTGTGCTCTCTATAGTGAATAGAGTTAGGCAGGGATACTCACCTTTGTCGTTGCAGACCCTTATCCCGAATCCAGGGGGACCCGACAGGCTCGGAAGAATCGAAGAAGAAGGTGGAGAGCAAGGCAGAGACAGATCAATTCGATTAGTGAACGGATTCTTAGCACTTGCCTGGGACGACCTGCGGAACCTGTGCCTCTTCAGCTACCACCGCTTGAGAGACTTTGTATTAGTGACAGCGAGAGTGGTGGAACTTCTGGGACGCAACAGCCTCAGGGGACTACAGAGGGGGTGGGAAGCCCTTAAATATCTGGGAAGTCTTGTGCAGTACTGGGGTCAGGAGCTAAAAAAGAGTGCTATTAGTCTGTTTGATACCATAGCAATAGTAGTAGCTGAAGGAACAGATAGGATTATARAAGCAGGACAAAGAYTTTRTAGAGCTATCTGCAACATACCTAGAAGAATAAGACAGGGCACTGAAGTAGTTTTGCAATAA

>BJ2015EU14

GCAGTGGGAATAGGAGCCATGATCTTTGGGTTCTTAGGAGCAGCCGGAAGCACTATGGGCGCAGCGTCATTAACGCTGACGGTACAGGCCAGACAATTATTGTCTGGTATAGTGCAACAGCAAAGCAATTTGCTGAGAGCTATAGAGGCGCAGCAGCATCTGTTGCAACTCACAGTCTGGGGCATTAAACAGCTCCAGGCAAGAGTCCTGGCTGTGGAAAGATACCTAAAGGATCAAAAGTTCCTAGGACTTTGGGGCTGCTCTGGAAGAATCATCTGCACCACTAATGTGCCCTGGAACTCCACTTGGAGTAACAAATCTTATGAAGAGATTTGGAACAACATGACATGGATAGAATGGGAGAAAGAAATTAGCAATTACACAAACAAAATCTATGACCTACTTACAGAATCGCAGAACCAGCAGGAAAGAAATGAAAAGGATTTGTTAGAGTTGGACAACTGGGCAAGCCTGTGGAATTGGTTTGAMATATCAAATTGGCTGTGGTATATAAGAATATTTATAATGATAGTAGGAGGGTTAATAGGTTTAAGAATAATTTTTGCTGTACTTTCTTTAGTAAATAGAGTTAGGCAGGGATACTCACCTTTGTCTTTTCAGACCCCTCCCCACCACCAGAGGGAACCCGACAGGCCCGAAGAAATCGAAGGAGAAGGTGGCGAGCAAGGCAGAGACAGATCCGTGAGATTAGTGAGCGGATTCTTAGCTCTTGCCTGGGACGATCTGCGGAGCCTGTGCCTCTTCAGCTACCGCCGCTTGAGAGACTTCATCTTGATTGCAACGAGGACTGTGGAACTTCTGGG------------CAAGGGACTGAGACGGGGGTGGGAGGGCCTCAAATATCTGGGAAATCTTCTGATATATTGGGGTCAGGAACTGAAAACTAGTGCTATCTCTTTGCTTGATGCTATAGCAATAACAACAGCGGAGTGGACAGATAGGGTTATAGAAGTTGCACAAAGAGTTTGGAGAGCTCTTCTCCACATACCTAGAAGAATCAGACAGGGCTTTGAAAGGGCTTTGCTATAA

>BJ2015EU15

GCAGTAACACTAGGAGCTGTGTTCCTTGGGTTCTTGGGAGCAGCAGGAAGCACTATGGGCGCAGCGTCAGTCACGCTGACGGTACAGGCCAGACAATTACTGTCTGGTATAGTGCAACAGCAGAACAATTTGCTGAAAGCTATTGAGGCGCAACAGCGTATGTTGCAACTCACAGTCTGGGGCATCAAGCAGCTCCAGGCAAGAGTCCTAGCTGTGGAAAGATACCTAAGGGATCAACAGCTCCTAGGGATTTGGGGTTGCTCTGGAAAACTCATCTGCACCACTAATGTRCCTTGGAATGCTAGTTGGAGTAATAGATCTTACMATGAAGTTTGGGACAACATGACCTGGATGCAGTGGGAGAGAGAAATTGACAATTACACAGGCTTAATATATGATTTACTTGAAAAATCGCAAAACCAGCAGGAAATCAATGAAAAGGAATTATTGGAGTTGGATAAGTGGGCAAGTTTGTGGAATTGGCTTGACATATCAAATTGGCTGTGGTACATAAAAATATTCATAATGATAGTAGGAGGCTTRATAGGTCTTAGAATAGTTTTTACTGTACTCTCTATAGTGAATAGAGTTAGGCAGGGTTACTCACCATTATCATTCCAGATCCGCCTCCCAGCTCAGAGGGGACCAGACAGGCCCGRAGGAATCGAAGAAGAAGGTGGAGAGAGAGACAGAGACACATCCGGGCGCTCAGTGACTGGATTCTTAGAACTCATCTGGGAAGACCTGTGGAACCTGTTCCGCTTCAGCTTCCTCCGCTTGAGAGACTTACTCTTGATTGTGACAAGGATTGTGGAACTTCTGGGACGCA---------------------GGGGGTGGGAAGCCCTCAAGTATTGGTGGAATCTCCTGCGGTATTGGAGTCAGGAACTAAAGAATAGTGCTATTAGCTTACTTAATGCCATAGCTATAACAGTAGCTGAGGGAACAGATAGAATTATAGAAGGAGCACAARGATTTGGTAGAGCTATTCTCAACATACCTAGAAGAATAAGACAGGGCTTAGAAAGGGCTTTGCTATAA

>BJ2015EU16

GCAGTGGGAATAGGAGCTATGATCTTTGGGTTCTTAGGAGCAGCAGGAAGCACTATGGGCGCGGCGTCAGTGACGCTGACGGTACAGGCCAGACAATTATTGTCTGGTATAGTGCAACAGCAAAGCAATTTGCTGAGGGCTATAGAGGCGCAACAGCATATGTTGCAACTCACAGTCTGGGGCATTAAACAGCTCCAGGCAAGAGTCCTGGCTGTGGAAAGATACCTAAAAGATCAAAAGTTCCTAGGACTTTGGGGCTGCTCTGGAAAAATCATCTGCACCACTGCTGTGCCCTGGAACTCCTCTTGGAGTAATAAAACTTATGAAGACATCTGGAACAACATGACATGGATAGAATGGGAGAGAGAAATTAGTAATTACACAGACACAATATACGAGTTGCTTGAAGACTCGCAGAACCAGCAGGACAGAAATGAAAAAGATTTGTTAGAATTGGACAAATGGAAAAACCTGTGGAATTGGTTTGACATAACAAATTGGCTGTGGTATATAAAAATATTCATAATGATAGTAGGAGGCTTGATAGGTTTAAGAATAATTTTTGYTGTGCTCTCTGTAGTGAATAGAGTTAGGCAGGGATACTCACCTTTGTCGTTGCAGACCCTTATCCCGAACCCAGGGGGACCCGACAGGCTCGGAGGAATCGAAGAAGAAGGTGGAGAGCAAGGCAGAGACAGATCCGTGCGATTAGTGAACGGATTCTTAGCACTTGCCTGGGACGACCTACGGAACCTGTGCCTCTTCTGCTACCACCGCTTGAGAGACTTTGTATTAGTGACAGCGAGAGTGGTGGAACTTCTGGGACGCAACAGCCTCAAGGGACTACAGAGGGGGTGGGAAGCCCTTAAATATCTGGGAAGYCTTGTGCAGTATTGGGGTCAGGAGCTAAAAAAGAGTGCTATTAGTCTGTTKGATACCATAGCAATAGCAGTAGCTGAAGGAACAGATAGGATTATAGAAGTAGGACAAAGAATTTGGAGAGCTATCTGCAACATACCTAGAAGAATAAGACAGGGCTTTGAAGCAGCTTTGCAATAA

>BJ2015EU17

GCAGTGGGAATAGGAGTTATGATCTTTGGGTTCTTAGGAGCAGCAGGAAGCACTATGGGCGCAGCGTCAATAGCGCTGACGGTACGGGCCAGACAATTATTGTCTGGTATAGTGCAACAGCAAAGCAATTTGCTGAGAGCTATAGAGGCGCAGCAGCATATGTTGCAACTCACAGTCTGGGGCATTAAACAGCTCCAGGCAAGAGTCCTGGCTGTGGAAAGATACCTAAAAGATCAAAAGCTCCTAGGCATTTGGGGCTGCTCTGGAAAAATCATCTGCACCACTGATGTGCCCTGGAACTCCAGTTGGAGTAATAAATCTTATTAAGAGATTTGGAACAACATGACATGGATAGAATGGGAGAGAGAAATTAGCAATTACACAAACAAAATATATGAATTACTTGAGGAATCGCAGAACCAGCAGGAAAGAAATGAAAAGGATTTGTTAGAATTGGACAAATGGGCAAGTCTGTGGAATTGGTTTGACATATCAAACTGGCTGTGGTATATAAAAATATTTATAATGATAGTAGGAGGGTTAATAGGTTTAAGAATAATTTTTGCTGTGCTTTCTATAGTAAATAGAGTTAGGCAGGGATACTCACCTTTATCTTTTCAGATCCCTTCCCATCATCAGAGGGAACCCGACAGGCCCGAAGGAATCGAAGAAGAAGGTGGCGAACGAGGCAGAGACAGATCCGTGAGATTAGTGAGCGGATTCTTAGCTCTTGCGTGGGACGATCTGCGGAGCCTGTGCCTCTTCAGCTACCGCCGCTTGAGAGACTTAATCTTGATTGCAACGAGGACTGTGGAACTTCTGGG------------CAAGGGACTGAGACGGGGGTGGGAAGGCCTCAAATATCTGGGGAATCTTCTGTTATATTGGGGTCAGGAACTGAAAACTAGTGCTATCTCTTTGCTTGATGCTATAGCAATAGCAACAGCGGGGTGGACAGATAGGGTAATAGAAGTTGCACAAAGAGCTTGGAGAGCTTTTCTCCACATACCGAGAAGAATCAGACAGGGCTTCGAAAGGGCTTTGCTATAA

>BJ2015EU19

GCAGTGGGAATAGGAGCTATGATCTTTGGGTTCTTGGGAGCAGCAGGAAGCACTATGGGCGCGGCGTCAATAACGCTGACGGTACAGGCCAGACAATTATTGTCTGGTATAGTGCAACAGCAAAGCAATTTGCTGAGGGCTATAGAGGCGCAGCAGCATATGTTGCAACTCACAGTCTGGGGCATTAAACAGCTCCAGGCAAGAGTCCTGGCTGTGGAAAGATACCTAAAGGATCAACAGTTCCTAGGACTTTGGGGCTGCTCTAACAAAATCATCTGCACCACTAATGTGCCCTGGAATACCTCTTGGAGTAATAAATCTTATGAAAAGATTTGGRACAACATGACATGGACAGAATGGGAGAGAGAAATTAGTAATTACACAAGCCAAATATATGAGTTGCTGATAGAATCGCAGAACCAGCAGGACAGAAATGAAAAGGATTTGTTAGAACTTGATAAATGGACAAGTCTGTGGAATTGGTTTGACATAACAAATTGGCTGTGGTATATAAAAATATTCATAATGATAGTAGGAGGCTTGATAGGKTTAAGAGTAATTTTTGCTGTGTGCTCTATAGTGAATAGAGTTAGGCAGGGATATTCACCTTTGTCGTTGCAGACCCTTATCCCGAACCCAGGGGGACCCGACAGGCTCGGAAGAATCGAAGAAGAAGGTGGAGAGCAAGGCAGAGACAGATCCGTTCGATTAGTGAACGGATTCTTAGCACTTGCCTGGGACGACCTGCGGAACCTGTGCCTCTTCAGCTACCACCGCTTGAGAGACTTTGTATTAGTGACAGCGAGAGTGGTGGAACTTCTGGGACGCAACAGCCTCAAGGGACTACGGAGGGGGTGGGAAGCCCTTAAATATCTAGGAAGTCTTGTGCAGTATTGGGGTCAGGAGCTAAAAAAGAGTGCTATTAGTCTGATTGATACTATAGCAATAGCAGTAGCTGAAGGAACAGATAGGATTATAGAAGTAGGACAAAGACTTTGTAGAGCTATCTACAACATACCTAGAAGAATAAGACAGGGCTTTGAAGCAGCTTTGCAATAA

>XC2014EU01

GCAGTGGGAATAGGAGCTATGATCTTTGGGTTCTTAGGAGCAGCAGGAAGCACTATGGGCGCGGCGTCAATAACGCTGACGGTACAGGCCAGACAATTATTGTCTGGTATAGTGCAACAGCAAAGCAATTTGCTGAGGGCTATAGAGGCGCAGCAGCATCTGTTGCAACTTACAGTCTGGGGCATTAAACAGCTCCAGGCAAGAATCCTAGCTGTGGAAAGATACCTAAAGGATCAAAAGTTCCTAGGACTTTGGGGCTGCTCTGGAAAAATCATCTGCACCACTGCTGTACCCTGGAACTCCACTTGGAGTAATAAATCCTTCGATGAGATTTGGAACAACATGACATGGATAGAATGGGAGAGAGAAATTAGCAATTACACAAAAACAATATTTGAGATACTTACAGAATCGCAAAACCAGCAGGACAAGAATGAACAAGAGTTGTTAGAGTTGGATAAATGGGCAAGTCTGTGGAATTGGTTTGACATAACAAAGTGGCTGTGGTATATAAAAATATTTATAATGATAGTAGGAGGTTTAATAGGTTTAAGAATAATTTTTGCTGTGCTTTCTATAGTGAATAGAGTTAGGCAGGGATACTCACCTGTGTCTTTCCAGACCCCTTTCCATCATCAGAAGGAACCCGACAGGCCCGAAARAATCGAAGAAGGAGGTGGCGAGCAAGGCAGCGACAGATCAGTGCGCTTAGTGAGCGGATTCTTAGCACTTATCTGGGACGATCTGCGGAGCCTGTGCCTCTTCAGCTACCACCGCTTGAGAGACTTCATCTTGGTGACARCGAGGATTGTGGAACTTCTGGGACACAGCAGTCTCAAGGGGCTGAGACGGGGGTGGGAAGGCCTCAAATATCTGGGGAATCTTCTGTTGTATTGGGGTCAGGAACTAAAAATTAGTGCTATTTCTTTGTTTGATGCTRCAGCAATAACARTAGCGGGGTGGACAGATAGGATTATCGAGCT------------TTGGAGAGCTATTTGCCACATACCTAGACGAATCAGACAGGGCTTAGAAAGGGCTTTGCTATAA

>XC2014EU05

GCAGTGGGACTAGGAGCTGTGCTCCTTGGGTTCTTGGGAGCAGCAGGAAGCACTATGGGCGCGGCGTCAATAACGCTGACGGTACAGGCCAGACAATTGTTGTCTGGAATAGTGCAACAGCAAAGCAATTTGCTGAGGGCTATAGAGGCGCAACAGCATATGTTGCAACTCACGGTCTGGGGCATTAAGCAGCTCCAGGCAAGAGTCCTGGCGATAGAAAGATACCTAAAGGATCAACAGCTCCTAGGGATTTGGGGCTGCTCTGGAAAACTCATCTGCACTACTGCTGTACCTTGGAACCTCAGTTGGAGTAACAAATCTCAAACAGAGATTTGGGATAACATGACCTGGATGCAGTGGGATAGAGAAATTAGTAATTACACAAACACAATATACAGGTTGCTTGAAGACTCGCAAAACCAGCAGGAAAAGAATGAAAAAGATCTATTAGCATTGGACAGTTGGAAAAATCTATGGAGTTGGTTTGACATAACAAATTGGCTGTGGTACATAAGAATATTCATAATAATAGTAGGAGGCTTAATAGGGTTAAGAATAATTTTTGCTGTGCTTTCTATAGTGAATAGAGTTAGGCAGGGATACTCACCTTTGTCGTTGCAGATCCCTACCCCGAACCCAGGGGGACCCGGCAGGCTCGGAAGAATCGAAGAAGGAGGTGGAGAGCAAGACAAAGACAGATCCATTCGATTAGTGAACGGATTCTTAGCACTTGCCTGGGACGACCTGCGGAACCTGTGCCTCTTCAGTTACCACCGATTGAGAGACTTCATATCAGTGACAGCGAGAGTGGTGGAACTTCTGGGACGCAGCAGCCTCAGGGGACTACAGAGGGGGTGGGAAGCCCTTAAATATCTGGGAAGTCTTGTGCAGTACTGGGGTCAGGAGCTAAAAAAGAGTGCTATTAGTCTAATTGATACCATAGCAATAACAGTAGCTGAAGGAACAGATAGGATTATAGAAGCAGCACAAGGACTTTGTAGAGCAATCTACAACATACCCAGAAGAATAAGACAGGGCTTTGAAGTAATTTTGCAATAA

>XC2014EU06

GCAGTG---GTAGGAGCTGTGTTCCTTGGGTTCTTGGGAGCGGCAGGAAGCACTATGGGCGCGGCGTCAATAACGCTGACGGTACAGGCCAGACAATTGCTGTCTGGTATAGTGCAACAGCAAAACAATTTGCTGAAGGCTATAGAGGCGCAACAGCATCTGTTGCAACTCACGGTCTGGGGCATTAAGCAGCTCCAGGCAAGAGTCCTGGCTATAGAAAGATACCTAAAGGATCAACAGCTCCTAGGGATTTGGGGCTGCTCTGGAAAACTCGTCTGCACTACTAATGTACCTTGGAACTCCAATTGGAGTAGCAAATCTCTAAATGAGATTTGGGATAACATGACCTGGATGCAGTGGGATAGAGAAATTAGTAATTACACAAACACAATATACGAGTTGCTTGAAGAATCACAAAACCAGCAGGAAAGGAACGAAAAAGATCTATTAGCATTGGACAGTTGGAAAAATCTATGGAATTGGTTTGACATATCAAATTGGCTGTGGTATATAAAAATATTTATAATAATAGTAGGAGGCTTGATAGGTTTAAGAATAATTTTTGCTGTGCTCTCTCTAGTGAATAGAGTTAGGCAGGGATACTCACCTTTGTCGTTTCAGATCCTTACCCCGAACCCAGGGGGACCCGGCAGGCCCGGAAGAATCGAAGAAGAAGGTGGAGAGCAAGACAAAGACAGATCCATTCGCTTAGTGAACGGATTCTTAGCACTTGCCTGGGACGACCTGCGAAACCTGTGCCTCTTCAGCTACCACCGCTTGAGAGACTTTGTATTAGTGACAGCGAGAGTGGTGGAACTTCTGGGACGCAACAGTCTCAGGGGACTACAGAGGGGGTGGGAAGCCCTTAAATATCTGGGAAGTCTTGTGCAGTACTGGGGTCAGGAACTAAAAAAGAGTACTGTTAGTCTGTTTGATACCATAGCAATAGCAGTAGCTGAAGGAACAGATAGGATTATAGAATTAGTACAAAGACTTTGTAGAGCTATCTACAACATACCTAGAAGAATAAGACAGGGCTTTGAAGCAGCTTTGCAATAA

>XC2014EU08

GCAGTAGGACTAGGAGCTGTGATCTTTGGGTTCTTGGGAGCAGCAGGAAGCACTATGGGCGCGGCGTCAATAGCGCTGACGGTACAGGCCAGACAATTGCTGTCTGGTATAGTGCAACAGCAAAGCAATTTGCTGAGGGCTATAGAGGCGCAACAGCATATGTTGCAACTCACGGTCTGGGGCATTAAGCAGCTCCAGACAAGAGTCCTGGCTATAGAAAGATACCTACAAGATCAACAGCTCCTAGGGATTTGGGGCTGCTCTGGAAAACTCATCTGCACCACTGCTGTACCTTGGAACTCCAGTTGGAGTAACAAATCTCAAGCAGAAATTTGGGATAACATGACTTGGATGCAGTGGGATAGGGAAATTAGTAATTACACATACACAATATACGGTTTGCTTGAAGATGCGCAAATCCAGCAGGAAAAAAATGAAAAGGATCTATTAGCATTGGACAGCTGGAAAAATCTTTGGACTTGGTTTGACATAACAAATTGGCTGTGGTACATAAAGATATTCATAATGATAGTAGGAGGCTTGATAGGTTTAAGAATAATCTTTGCTGTGCTCTCTATAGTGAATAGAGTTAGGCAGGGATACTCACCCTTGTCGTTGCAGACCCTTACCCCGAACCCAGGGGGACCCGACAGGCTCGGAGGAATCGAAGAAGGAGGTGGAGAGCAAGACAACGCCAGATCCATTCGATTAGTGAACGGATTCTTAGCACTTGCCTGGGACGACCTGCGGAGCCTGTGCCTCTTCAGCTACCACCGATTGAGAGACTTAATATTAGTGACAGCGAGGGTGGTGGAACTTCTGGGACGCAGCATTCTCAGGGGACTACAGAGGGGGTGGGAAGCCCTGAAATATCTGGGGAGTCTTGTGCAGTACTGGGGTCAGGAGCTRAAAAAGAGTGCTATTAGYCTGCTTGATACCACAGCAATAATAGTAGCTGAAGGAACAGATAGGATTYTAGAARTAGTACAAGGATTTTGTAGAGCTATATACAACATACCTAGAAGAATAAGACAGGGCTTTGAAGCAGCTTTGCAATAA

>XC2014EU09

GCAGTGGGAATAGGAGCTGTGTTCCTTGGGTTCTTGGGAGTAGCAGGAAGCACTATGGGCGCGGCGTCAATGGCGCTGACGGGACAGGCCAGACAATTGCTGTCTGGTATAGTGCAACAGCAAAACAATTTGCTGAGGGCTATAGAGGCGCAACAGCATATGTTGCAACTCACGGTCTGGGGCATTAAACAGCTCCAAACAAGAGTCCTGGCTATAGAAAGATACCTAAAGGATCAACAGCTCCTAGGAATTTGGGGCTGCTCTGGAAAACTCATCTGCCCCACTGCTGTACCTTGGAACTCCAGTTGGAGTAACAAATCTCAATCAGAGATTTGGGATAACATGACCTGGATGCAATGGGATAAAGAAATTAGTAATTACACAGACATAATATACAAGTTGCTTGAAGTCTCGCAAAACCAGCAGGAAAGGAATGAGAAAGATCTATTAGCATTGGACAGTTGGAAAAATCTATGGAATTGGTTTAACATAACAAACTGGCTGTGGTACATAAAAATATTCATAATGATAGTAGGAGGCTTGATAGGTTTAAGAATAATTTTTGCTGTGCTTTCTATAGTGAATAGAGTTAGGCAGGGATACTCACCTTTGTCGTTGCAGACCCTTATCCCGAACCCAAGGGGACCCGACAGGCCCGGAGGAATCGAAGAAGAAGGTGGCGAGCAAGGCAGAGACAGATCCGTGCGATTAGTGAGCGGATTCTTTGCTCTTGCCTGGGACGACCTGCGGAGCCTGTGCCTCTTCAGCTACCGCCGCTTGAAAGACTTCATCTCAATTGCAACGAGGACTGTGGAACTTCTGGG------------CAGGGGACTGAGACGGGGGTGGGAAGGCCTCAAATATCTGGGGAGTCTTCTGTTGTATTGGAGTCGGGAGCTGAAAATTAGTGCTGTTTCCTTGATTGATACTTTAGCAATAACAATAGCGGGGTGGACAGATAGGGTCATAGAAGTAGCACAAAGAGCTTGGAGAGCTATTCTCCACATACCTAGAAGAATTAGACAGGGCTTCGAAAGGGCTTTGCTATAA

>XC2014EU10

GCAGTGGGAATAGGAGCTGTGTTCCTTGGGTTCTTGGGAGCAGCAGGAAGCACTATGGGCGCGGCGTCAGTGGCGCTGACGGTACAGGCCAGGCAATTGCTGTCTGGTATAGTGCAACAGCAAAGCAATTTGCTGAGGGCTATAGAGGCGCAACAGCATATGTTGCAACTCACGGTCTGGGGCATTAAACAGCTCCAGGCAAGAGTCCTGGCTATGGAAAGATACCTAAAGGATCAACAGCTCCTAGGGATTTGGGGCTGCTCTGGAAAACTCATCTGCACCACTGCTGTACCTTGGAACTCCAGTTGGAGTAACAAATCCAAAGATGAGATTTGGGGGAACATGACCTGGATGCAGTGGGATAGGGAAATTAGTAATTACACAGGCATAATATACAGGTTGCTTGAAGACTCGCAAAACCAGCAGGAAAGAAATGAAAAAGATCTATTAGCATTGGACAGTTGGAAAAATCTATGGAGTTGGTTTGACATAACAAATTGGCTGTGGTATATAAAAATATTCATAATAATAGTAGGAGGCTTGATAGGTTTAAGAATAATTTTTGCTGTGCTTTCTATAGTGAATAGAGTTAGGCAGGGATACTCACCTCTGTCGTTGCAGACCCTTATCCCGAACCCAGGGGGACCCGACAGGCTCGGAAGAATCGAAGAAGAAGGTGGAGAGCAAGACAAAGACAGATCCATTCGATTAGTGAACGGATTCTTTGCGCTTGCCTGGGACGACCTGCGGAACCTGTGCCTCTTCAGCTACCACCGCTTGAGAGACTTAATATTAGTGACAGCGAGAGTGGTGGAACTTCTGGGACGCAGCAGTCTCAGGGGACTACAGAGGGGGTGGGAAACCCTTAAATATCTGGGAAGTCTTGCGCAGTGCTGGGGTCAGGAGCTAAAAAGGAATATTACTAGTCTGATTGATACCCTAGCAATAGTAGTAGCTGAAGGAACAGATAGGATTATAGAAGTAGTACAAAGATTTTGTAGAGCTATCTACAACGTACCTAGAAGAATAAGACAGGGCTTGGAAGCAGCTTTGCAATAA

>XC2014EU13

GCAGTAGGAATAGGAGCTGTGTTCCTTGGGTTCTTGGGAGCAGCAGGAAGCACTATGGGCGCAGCGTCAATAACGCTGACGGTACAGGCCAGACAATTGCTGTCTGGTATAGTGCAACAGCAAAGCAATTTGCTGAGGGCTATAAAGGCGCAACAGCATCTGTTGCAACTCACGGTCTGGGGCATTAAGCAGCTCCAGACAAGAATCCTGGCTATAAAAAGATACCTAAAGGATCAACAGCTCCTAGGGATTTGGGGCTGCTCTGGAAAGCTCATCTGCCCTACTGCTGTACCTTGGAACTCCAGTTGGAGTAATAAAACTTACAATGAGATTTGGGATAACATGACCTGGATGCAGTGGGATAGAGAAATTAGTAATTACACAGACACAATATACAGATTGCTTGAAGACTCGCAAAACCAGCAGGAAAGGAATGAAAAAGACCTATTGGCATTGGACAGTTGGAAGAATCTATGGAGTTGGTTTAACATAACAAATTGGCTGTGGTATATAAGAATATTCATAATAATAGTAGGAGGATTGATAGGTTTAAGAATAATTTTTGCTGTGCTTTCTATAGTGAATAGAGTTAGGCAGGGATACTCACCTTTGTCATTGCAGACCCTTATCCCGAACCCAGTGGGACCCGACAGGCCCGGAGGAATCGAAGAAGGAGGTGGAGAGCAAGGCAGAGACAAATCAATTCGATTAGTAACCGGATTCTTAGCACTTGCCTGGGACGACCTGCGGAACCTGTGCCTCTTCAGCTACCACCAATTGAGAGACTTAATATTAGTGACAGCGAGAGTGGTGGAACTTCTGGGACACCACAGTCTCAAGGGACTACAGAGGGGGTGGGAAGCCCTTAAATATCTGGGAAGTCTTGTGCAGTACTGGGGCCAGGAGCTAAAAAAGAGTGCTATTAGTCTGCTTGATACCATAGCAATAGCAGTAGCTGCAGGAACAGATAGGATTATAGAAGTATTTCAAAGACTTGGTAGAGCCATCTGCAACATACCTAGAAGAATAAGACAGGGCCTTGAAGCAGCTTTGCAATAA

>XC2014EU18

GCAGTGGGAATAGGAGCTATGATCTTTGGGTTCTTAGGAGCAGCAGGAAGCACTATGGGCGCAGCGTCAATAACGCTGACGGTACAGGCCAGACAATTATTGTCTGGTATAGTGCAACAGCAAAGCAATTTGCTGAGAGCTATAGAGGCGCAGCAGCATCTGTTGCAACTCACAGTCTGGGGCATTAAACAGCTCCAGGCAAGAGTCCTGGCTGTGGAAAGATACCTAAAAGATCAAAAGTTCCTAGGACTTTGGGGCTGCTCTGGAAAAATCATCTGCACCACTAATGTGCCCTGGAACGAAACTTGGAGTAATAAATCCTTAAAGGAGATTTGGGACAACATGACATGGACAGAATGGGAGAGAGAAATTAGCAATTACACAAACATAATCTATGAGCTACTTACAGAATCGCAGAACCAGCAGGACAAAAATGAAAAGGAGTTGTTAGAGTTTGACAAATGGGCAAGTCTGTGGAATTGGTTTGACATAACAAGATGGCTGTGGTATATAAAAATATTTATAATGATAGTAGGAGGTTTAATAGGTTTAAGAATAATTTTTGCTGTGCTTTCTATAGTAAATAGAGTTAGGCAGGGATACTCACCTTTGTCTCTCCAGACCCCTACCCATCAGCAGAGGGAACCCGACAGGCCCGAAGGAACCGAAGAAGAAGGTGGAGAGCAAGGCAGAGACAGATCCGTGCGATTAGTGAGCGGATTCTTATCACTTATTTGGGACGACCTACGGAGCCTGTGCCTCTTCAGCTACCACCACTTGAGAGACTTCATCTCAATTGCAGCGAGGACTGTAGAACTTCTGGGACGCTGCAGTCTCAAGGGACTGAGACGGGGGTGGGAAGGCCTCAAATATCTGGGGAATCTGCTGTTATATTGGGGTCAGGAACTAAAAACTAGTGCTATTTCTTTGCTTGATACTATAGCAATAGCAGTAGCGGGGTGGACAGATAGGGTTATAGAAGTAGCACAAAGAACTGGGAGAGCTATCCTCCACATACCTAGAAGAATCAGACAGGGCTTAGAAAGGGCTTTGCTATAA

>XC2014EU19

GCAGTGGGACTAGGAGCTGTGTTCCTTGGGTTCTTGGGAGCAGCAGGAAGCACTATGGGCGCGGCGTCAATAACGCTGACGGTACAGGCCAGACAACTGTTGTCTGGTATAGTGCAACAGCAAAGCAATTTGCTGAAGGCTATAGAGGCGCAACAGCATCTGTTGCAACTCACGGTCTGGGGCATTAAACAGCTCCAGACAAGAGTCCTGGCTATAGAAAGATACCTAAAGGATCAACAGCTCCTAGGGATCTGGGGCTGCTCTGGAAAACTCATCTGCACCACTGCTGTACCTTGGAACTCCAGTTGGAGTAACAAATCTCACGACGAGATTTGGAATAATATGACCTGGATGCAATGGGATAGAGAAATTAATAATTACACAAACACAATATACAGGTTGCTTGAAGAATCACAAAACCAGCAGGAAAAGAATGAAGAGGATCTATTAGCATTGGACAGTTGGAACAATCTATGGAGTTGGTTTAATATAACACATTGGCTGTGGTATATAAGAATATTCATAATGATAGTAGGAGGCTTGATAGGTTTAAGAATAATTTTTGCTGTGCTCTCTATAGTGAATAGAGTTAGGCAGGGATACTCACCTGTGTCGTTTCAGATCCCTACCCCGAACCCAGGGGGACCCGAGCGGCTCGGAAGAATCGAAGAAGAAGGTGGAGAGCAAGACAAAGACAGATCCATTCGCTTAGTGAACGGATTCTTTGCACTTGCGTGGGACGATCTACGGAGCCTGTGCCTCTTCAGCTACCACCGATTGAGGGACTTCATATTAGTGACAACGAGAGTGGTGGAACTTCTGGGACGCAGCAGCCTCAGGGGACTACAGAGGGGGTGGGAAGTCCTTAAATATCTGGGAAGTCTTGTGCAGTACTGGAGTCAGGAGCTAAAAAAGAGTGCTATTAGTCTGATTGATACCCTAGCAATAGCAGTAGGTGAAGGAACAGATAGGATTCTAGAATTAGTACAAAGGCTTGGTAGAGTTATCTACAACATACCTACAAGAATAAGACAGGGCCTTGAAGCAGCTTTGCAATAA

>XC2014EU20

GTAGTGGGAATAGGAGCTATGTTCCTTGGGGTCTTGTCGGCAGCAGGAAGCACTATGGGCGCGGCGTCAATAACGCTGACGGTACAGGCCAGACAATTGCTGTCTGGTATAGTGCAACAGCAAAGCAATTTGCTGAGAGCTATAGAGGCGCAACAGCATCTGTTGCAACTCACGGTCTGGGGCATTAAACAACTCCAGGCAAGAGTCCTGGCTATAGAAAGATACCTAAAGGATCAACAGCTCCTAGGGATTTGGGGCTGCTCTGGAAAACTCATCTGCACTACTGCTGTACCTTGGAACTCCAGTTGGAGTAATAAATCTCAAACAGAGATTTGGGATAACATGACCTGGATGCAGTGGGATAAGGAAATTAGTAACTACACAAACACAATATACAGTCTGCTTGAAGACTCACAAAAACAGCAGGAAAAAAATGAAAAGGATTTATTAGCATTGGACAGTTGGAATAATCTATGGAATTGGTTTGACATATCAAAATGGCTGTGGTATATAAAAATATTCATAATAATAGTAGGAGGCTTGATAGGCTTAAGAATAATTTTTGCTGTGCTCTCTATAGTGAATAGAGTTAGGCAGGGATACTCACCTTTGTCGTTCCAGACCCCGATCCCGAACCCAGGGGGACTCGACAGGCTCGGAAGAATCGAAGAAGAAGGTGGAGAGCAAGACAACGCCAGATCCATTCGATTAGTGAACGGATTCTTAGCACTTTTCTGGGACGACCTCCGGAACCTGTGCCTCTTCAGCTACCACCGCTTGAGAGACTTACTATTACTGATAGCGAGAGGAGTGGAACTTCTGGGACGCAGCAGCCTCAAGGGACTACAGAGGGGGTGGACAGCCCTTAAATATCTGGGAAATCTTGTGCAGTATTGGGGTCTGGAGCTAAAAAAGAGTGCTAGTAGTCTGTTTGATAGCATAACAATAGCAGTAGCTGAAGGGACAGATAGGATTATAGAAGTAGTACAAGGATTTTGTAGAGCTATCTACAATATACCTACAAGAATAAGACAGGGCCTTGAAGCAGCTTTGCAATAA
